# Identification of immune subsets with distinct lectin binding signatures using multi-parameter flow cytometry: correlations with disease activity in systemic lupus erythematosus

**DOI:** 10.3389/fimmu.2024.1380481

**Published:** 2024-05-07

**Authors:** Enikő Szabó, Anna Faragó, Gergely Bodor, Nikolett Gémes, László G. Puskás, László Kovács, Gábor J. Szebeni

**Affiliations:** ^1^ Institute of Genetics, Laboratory of Functional Genomics, HUN-REN Biological Research Center, Szeged, Hungary; ^2^ Core Facility, HUN-REN Biological Research Centre, Szeged, Hungary; ^3^ Astridbio Technologies Ltd, Szeged, Hungary; ^4^ Doctoral School of Multidisciplinary Medical Sciences, Albert Szent-Györgyi Medical School, University of Szeged, Szeged, Hungary; ^5^ Department of Rheumatology and Immunology, Albert Szent-Gyorgyi Medical School and Health Center, University of Szeged, Szeged, Hungary; ^6^ Department of Internal Medicine, Hematology Center, Faculty of Medicine, University of Szeged, Szeged, Hungary

**Keywords:** systemic lupus erythematosus, flow cytometry, glycobiology of SLE, lectin binding, immunophenotyping

## Abstract

**Objectives:**

Cell surface glycosylation can influence protein-protein interactions with particular relevance to changes in core fucosylation and terminal sialylation. Glycans are ligands for immune regulatory lectin families like galectins (Gals) or sialic acid immunoglobulin-like lectins (Siglecs). This study delves into the glycan alterations within immune subsets of systemic lupus erythematosus (SLE).

**Methods:**

Evaluation of binding affinities of Galectin-1, Galectin-3, Siglec-1, *Aleuria aurantia* lectin (AAL, recognizing core fucosylation), and *Sambucus nigra* agglutinin (SNA, specific for α-2,6-sialylation) was conducted on various immune subsets in peripheral blood mononuclear cells (PBMCs) from control and SLE subjects. Lectin binding was measured by multi-parameter flow cytometry in 18 manually gated subsets of T-cells, NK-cells, NKT-cells, B-cells, and monocytes in unstimulated resting state and also after 3-day activation. Stimulated pre-gated populations were subsequently clustered by FlowSOM algorithm based on lectin binding and activation markers, CD25 or HLA-DR.

**Results:**

Elevated AAL, SNA and CD25^+^/CD25^-^ SNA binding ratio in certain stimulated SLE T-cell subsets correlated with SLE Disease Activity Index 2000 (SLEDAI-2K) scores. The significantly increased frequencies of activated AAL^low^ Siglec-1^low^ NK metaclusters in SLE also correlated with SLEDAI-2K indices. In SLE, activated double negative NKTs displayed significantly lower core fucosylation and CD25^+^/CD25^-^ Siglec-1 binding ratio, negatively correlating with disease activity. The significantly enhanced AAL binding in resting SLE plasmablasts positively correlated with SLEDAI-2K scores.

**Conclusion:**

Alterations in the glycosylation of immune cells in SLE correlate with disease severity, which might represent potential implications in the pathogenesis of SLE.

## 1 Introduction

SLE is a complex autoimmune disease characterized by immune system dysregulation, particularly the loss of tolerance to nucleic acids and nucleic acid binding proteins initially described in B-cells, but later nuclear antigen specific T-cells have also been discovered (1). The differentiation and expansion of the pathogenic T-cell subsets is linked with the release of type I interferons (IFN-I), (2, 3) the fundamental contributors to SLE pathogenesis. IFN-I are released predominantly by plasmacytoid dendritic cells, neutrophil granulocytes, monocytes, and certain epithelial cells in response to engagement with nucleic acid-containing immune complexes or cytoplasmic nucleic acids. IFN-I induce the transcription of hundreds of genes playing role in antiviral activity and immune function regulation, thereby promoting both innate and adaptive immune cell functions (4, 5). Although autoantibody-producing B cells and CD4 helper T-cells are key upstream drivers of SLE, extensive research unraveled the importance of various immune subsets contributing to the clinical picture observed in SLE recently reviewed by others (6–8). The intricate interplay among these immune cells, coupled with genetic and environmental factors, contributes to the challenging and multifaceted nature of SLE.

Fueled by the recent advancements in the field of multi-parametric flow cytometry and mass cytometry, simultaneous complex immune phenotyping has become possible bringing new perspectives on understanding SLE. To tackle the complexity of high-dimensional data, novel bioinformatics tools has been developed enabling the identification of unique cell subsets that surpass the limitations of conventional manual gating. Many algorithms have been devised for automated population identification using clustering (9). An outstanding example known for its strong performance and computational efficiency is FlowSOM (Flow self-organizing map), which adapts the concept of self-organizing maps to cytometry data (10). Studies utilizing FlowSOM unveiled the intrinsic heterogeneity within populations of particular interest in SLE, like peripheral helper T-cells and CD11c^+^ B-cells, both reportedly expanded in SLE linked with disease severity (11–13).

As of now, the clustering is primarily grounded in the expression of proteins on the cell surface or the intracellular space. However, posttranslational modifications of proteins such as glycosylation have been reported to influence protein-protein interactions. The most relevant alterations of glycans of the immune system include changes in glycan complexity, fucosylation, and terminal sialylation status highlighted here through a few - admittedly selective - examples. Core fucosylation is essential for the activation of T-cell receptor (TCR), (14) antigen recognition and signal transduction via B-cell receptor (BCR), (15) and also for the expression of programmed cell death receptor-1 (PD-1) (16). Sialic acids are the ligands of Siglecs, a family of receptors with immunoregulatory function. Siglecs play a crucial role in distinguishing between self antigens and pathogens, since sialic acids are usually absent in microbes but present on higher vertebrates (17). Alpha-2,6-sialylation on self-antigens contributes to peripheral B-cell tolerance through the binding to inhibitory co-receptors Siglec-2 and Siglec-10 of BCR (18). Alpha-2,6-sialic acid also affects the binding of galectins (Gals), another family of carbohydrate binding proteins, either completely hampering or reducing glycan binding (19). Galectins are multivalent beta-galactoside binding proteins secreted by various cell types and tissues including immune cells, thus galectins are involved in wide range of immunological processes. Gal-1, Gal-3 and Gal-9 are the most abundantly expressed by immune cells exerting immunosuppressive effects in the adaptive immune system. Gal-1 and Gal-3, in particular, have been both shown to trigger apoptosis of T-cell subsets through distinct mechanisms, (20) and they are also known to regulate BCR signaling, as well as differentiation and survival of plasma cells (21). Terminal alpha-2,6-sialylation in T helper type 2 (Th2) and regulatory T-cells (Treg) has been demonstrated to confer protection against Gal-1 induced apoptosis compared to other T-cell subsets (22, 23). In the context of SLE, increased serum Gal-1 (24), Gal-3 (25) and Gal-9 (26) was reported. Markovic et al. proposed that, during systemic inflammation Gal-1 is released to prevent further tissue damage and destruction, and to counter-affect the pro-inflammatory cytokines (27). However, it is still unclear why this elevated level of immunoregulative Gals does not seem to alleviate systemic inflammation in SLE. One possible explanation is the reduced availability of glycan structures on the surface of target cells needed for galectin binding. Therefore, we underscore the importance of studying the ability of both Gal-1 and Gal-3 to bind to target immune cells.

In SLE, diverse alterations in N-glycosylation have been identified in both total immunoglobulin G (IgG) and IgG specific to anti-ds-DNA, and many of them are associated with the severity of the disease. However, cell surface glycosylation of immune cells in human SLE is relatively understudied and solely focuses on unstimulated immune cells (28). Notwithstanding, it has long been established that activation induces substantial changes in glycosylation of mouse T- and B-lymphocytes (29, 30) and glycosylation has been demonstrated to impact effector functions as well (14, 15, 31, 32). Hence, it is indubitably valuable to study the glycosylation of SLE immune cells during “ex vivo” stimulation mimicking the dysregulated immune response in SLE. Accordingly, our previous research highlighted a reduced sensitivity of activated SLE T-cells to Gal-1 induced apoptosis potentially linked to higher degree of alpha-2,6-sialylation in activated state compared to healthy controls (33, 34).

The present study aimed to expand our understanding of immune cell glycosylation in SLE by applying multi-parameter flow cytometry to monitor key immunologically relevant glycosylation changes, specifically, alpha-2,6-sialylation and core fucosylation by the binding of SNA and AAL, respectively. Furthermore, the binding of endogenous immunoregulatory lectins, Gal-1, Gal-3 and Siglec-1 was assessed. The plasma level of soluble Siglec-1 is reportedly a biomarker of renal involvement in SLE (35). The binding of the aforementioned lectins were quantified across subsets of T-cells, NK-cells, NKT-cells (first panel), and B-cells and monocytes (second panel). The measurements were carried out before (regarded as resting state) and after 3 days of stimulation by polyclonal T-cell activator (CytoStim) and recombinant IL-2 for the first panel or by LPS and TLR9 agonist (ODN2006) for the second panel. To monitor the level of activation, activation-induced markers were used: CD25 for the first panel or HLA-DR for the second panel. CD25 is part of the IL-2 receptor complex and highly expressed in activated circulating immune cells and Tregs (36). In contrast, HLA-DR is expressed constitutively by B-cells and monocytes, albeit its expression is upregulated in response to stimulation by CpG (ODN2006) in B-cells or by LPS in monocytes (37, 38). To our knowledge, this study marks the first instance utilizing FlowSOM to cluster immune cell subsets of stimulated PBMCs in SLE based on lectin-binding and activation marker expression (CD25 for the first panel and HLA-DR for the second panel). Herein, we present significant changes in lectin binding between PBMC subsets of SLE subjects and healthy individuals, as well as altered abundance of subtypes within major immune cell populations in SLE compared to controls revealed by FlowSOM. Of special highlight, many of these parameters show significant correlation with disease activity.

## 2 Materials and methods

### 2.1 Ethical statement

Patients were recruited during visits at the Department of Rheumatology and Immunology (University of Szeged). Healthy controls were voluntary employees of the BRC or University of Szeged. Subjects were informed about the research by a physician. Written informed consent was obtained from all subjects, and our study was reviewed and approved by an independent ethical committee of the university. Details about the study design and handling of biological materials were submitted to the Human Investigation Review Board of the University of Szeged under the 21/2011 and 149/2019-SZTE Project Identification codes. Laboratory studies and interpretations were performed on anonymized samples lacking personal identifiers. The study adhered to the tenets of the most recent revision of the Declaration of Helsinki.

### 2.2 Patients

Adult SLE patients meeting the 2019 European League Against Rheumatism/American College of Rheumatology classification criteria were included if they had active disease (either newly diagnosed or relapsing) as defined as at least one BILAG A or at least two BILAG B scores (39) or a SLEDAI-2K score ≥ 6 (40, 41). Patients with co-morbid malignant disease, diabetes mellitus, end-stage renal disease, a concurrent other inflammatory condition (e.g. infection) or overlapping systemic autoimmune disease except antiphospholipid syndrome or Sjögren’s syndrome with clinically active extraglandular manifestations were excluded. Newly diagnosed patients were therapy-naive, whereas relapsing patients were on maintenance immunosuppressive therapy, but corticosteroid dose > 5 mg/day prednisolone resulted in exclusion. Healthy controls were age-and sex-matched individuals who did not have a current or past chronic inflammatory disease (autoimmune or other immune-mediated), or a current acute inflammatory (infectious or non-infectious) disease.

Demographic data, anti-dsDNA levels, SLEDAI- 2K activity indices, and the distribution of clinical domains are presented in **Supplementary Table 1**. The number of patients was different in the two panels. From the T-cell/NK-cell/NKT-cell panel, 5 samples (and their paired controls) had been excluded due to methodological reasons, while for the B-cell/monocyte panel, 18 samples were available. Age, serological and clinical activity of the patients included in the T-cell/NK-cell/NKT-cell panel are presented in **Supplementary Table 2**. There was no significant difference in the studied clinical parameters between this group and the whole cohort. Demographic data of the control cohort is summarized in **Supplementary Table 3**.

### 2.3 PBMC isolation

PBMCs were isolated as described previously by our group (42, 43). Briefly, after the collection of 20 mL peripheral blood into an EDTA vacutainer (Becton Dickinson, Franklin-Lakes, USA), PBMCs were isolated by standard density gradient centrifugation using Leucosep tubes (Greiner Bio-One, Kremsmünster, Austria). Residual red blood cells were lysed suspending the PBMC pellet in 2 mL Ammonium-Chloride-Potassium Lysis Buffer (ACK: 0.15 M NH_4_Cl, 10 mM KHCO_3_, 0.1 mM Na_2_EDTA, pH 7.3; Merck, Darmstadt, Germany) and incubating at room temperature (RT) for 2 min. Samples were washed twice with 10 mL phosphate-buffered saline (PBS, Merck), and subsequently, cell count and viability was determined with trypan blue (Merck) exclusion test. Aliquots of 4 × 10^6^ PBMCs were subjected to cryopreservation in Fetal Bovine Serum (FBS, Capricorn Scientific, Ebsdorfergrund, Germany) containing 10% (v/v) dimethyl sulfoxide (DMSO, Merck) in liquid nitrogen (Messer, Bad Soden, Germany).

### 2.4 Lectin conjugation with fluorophores

For covalent labeling of lectins with fluorophores targeting the primary amine groups of lectins, Lightning Link conjugation kits were used following the simple and rapid procedure provided by the manufacturer (Abcam, Cambridge, US). *Aleuria Aurantia* lectin (AAL, Cat. L-1390-2, Vector Laboratories, Newark, USA) was labeled with APC (Cat. ab201807, Lot.GR3388854-1), recombinant human galectin-1 as a gift of Dr. Éva Monostori, Gal-1, expression and purification steps described in detail in (44–46), that was conjugated to PE/Cy7 (Cat. ab102903, Lot. GR3390407-1), recombinant human sialoadhesin, Siglec-1/CD169 protein (Siglec-1, Cat. 5197SL050, Fischer Scientific, Massachusetts, USA) was labeled with PE/Texas Red (Cat. ab269899, Lot. GR3395603-2), and finally galectin-3 (Gal-3, Cat. 450-38, PeproTech, London, UK) was chemically linked to APC/Cy7 dye (Cat. ab102859, Lot. GR3396716-1). Fluorescein-conjugated *Sambucus nigra* agglutinin was purchased from Vector Laboratories (Cat. FL-1301-2). The list of the selected lectins for cytometry is listed in **Supplementary Table 4**.

### 2.5 Flow cytometry protocol for T-cells, NK-cells and NKT-cells

Flow cytometry was performed as described previously by our group with some modifications (47, 48). Briefly, one aliquot of PBMC per patient was thawed and plated onto a flat-bottomed 48-well plate (Biologix, Hallbergmoos, Germany). The samples were divided into day O and day 3 sample referred as “resting state” and “activated state”, respectively. Both samples were incubated for 2 hours at 37°C in complete RPMI media (Capricorn Scientific) supplemented with 10% FBS (200 μL/well). After two hours, the stimulating CytoStim (100x dilution, Cat. 130-092-172, Miltenyi Biotec, Auburn, USA) reagent was added in complete RPMI to the “activated state” samples along with recombinant human IL-2 (10 ng/mL, Cat. CYT-209, ProSpec, Rehovot, Israel) in a final volume of 400 μL per well and these samples were housed for 72 hours inside a standard tissue culture incubator (37°C, 5% CO_2_, humidified). The unstimulated “resting state” samples were labelled in the following steps. Samples were transferred to FACS tubes, the wells were washed twice with PBS containing 2 mM EDTA. The cells were washed once with 1 mL PBS and centrifuged at 370 g for 6 min. After discarding the supernatant, cells were resuspended in 50 μL PBS containing human TruStain FcX Fc receptor blocking reagent (25x dilution, Cat. 422302, BioLegend, San Diego, USA) and Viobility 405/520 fixable viability dye (100x dilution, Cat. 130-130-404, Miltenyi Biotec). Incubation at room temperature 15 minutes followed by washing with 1 mL PBS, and centrifugation 370 g for 6 minutes. Prior to fixation, cell pellets were re-suspended in 100 μL EDTA FACS buffer (PBS +1% FBS+0.1% sodium azide + 2 mM EDTA), then each sample received 100 μL of 3.5% formaldehyde solution and were mixed by pipetting and incubated at room temperature for 20 minutes. After fixation, the samples were washed twice with 1 mL PBS and centrifuged at 500 g for 7 minutes. Cells were stained with lectin and antibody cocktails in 100 μL PBS per sample on ice for 30 minutes. Dilution of antibodies: anti-human CD3 efluor450 (Cat. 48-0038-42, clone: UCHT1, Thermo Fisher Scientific, Waltham, Massachusetts, USA) 80x; anti-human CD4 Alexa Fluor 700^®^ (Cat. 317426, clone: OKT4, BioLegend) 80x; anti-human CD8 PerCP (Cat. MHCD0831, clone: 3B5, Thermo Fisher Scientific) 80x; anti-human CD56 BV605 (Cat.562780, clone: NCAM16.2, Beckton Dickinson, Franklin Lakes, USA) 80x; anti-human CD25 Super Bright™ 645 (Cat. 64-0259-42, clone: BC96, Thermo Fisher Scientific) 40x. Lectin concentration: AAL 0.1 μg/mL; Gal-1 0.2 μg/mL; Gal-3 2.5 μg/mL; SNA 1.25 μg/mL; Siglec-1 0.5 μg/mL. Staining was followed by a single washing step with 1 mL FACS buffer and cells were collected by centrifugation at 500 g for 7 minutes. Cell pellets were resuspended in 300 µL FACS Buffer and measured on CytoFLEX S V4-B2-Y4-R3 Flow Cytometer (Cat. C09766, Beckman Coulter, Indiana, USA). At the endpoint of 72-hour activation, the same staining protocol was repeated with the “activating state” samples. Examples of compensations avoiding of signal spillover for the antibody panel are attached as: **Supplementary Material** Compensation_Histograms.

### 2.6 Flow cytometry protocol for B-cells and monocytes

The procedure was the same as the measurement of the T-cells/NK-cells and NKT-cells above, with the following modifications. First, in case of “activated state” samples, lipopolysaccharide (100 ng/mL, Cat. L2880, Sigma-Aldrich, Saint Louis, USA) and TLR9 ligand/agonist/ODN2006 (0.8 μM, 5’-tcgtcgtttgtcgttt-3’) were used in combination for activation of B-cells and monocytes within the total PBMC for 72 hours in complete RPMI medium. Second, different antibodies were used for staining B-cells and monocytes. Dilution of antibodies: anti-human CD19 BV605 (Cat. 363024, clone: SJ25C1, BioLegend) 80x; anti-human CD14 BV650 (Cat. 301836, clone: M5E2, BioLegend) 40x; anti-human CD16 Alexa Fluor 700^®^ (Cat. 302026, clone: 3G8, BioLegend) 50x; anti-human CD27 Pacific Blue™ (Cat. 302822, clone: O323, BioLegend) 100x; anti-human CD38 PerCP (Cat. 356622, clone: HB-7, BioLegend) 40x; anti-human HLA-DR (Cat. 14-9956-82, clone: LN3, Thermo Fisher Scientific) 800x. HLA-DR antibody was conjugated with Nova Fluor™ Yellow 690 antibody labelling kit (Cat. K06T04L014, Thermo Fisher Scientific). The latter Nova Fluor™ fluorophore required the addition of 5 μL/sample CellBlox™ Blocking Buffer (Cat. B001T03F01, Thermo Fisher Scientific) into the staining cocktail. The concentration of lectins applied for labelling the cells were same as in the above protocol. Examples of compensations avoiding of signal spillover for the antibody panel are attached as: **Supplementary Material** Compensation_Histograms.

### 2.7 Data analysis

**Figure 1:**
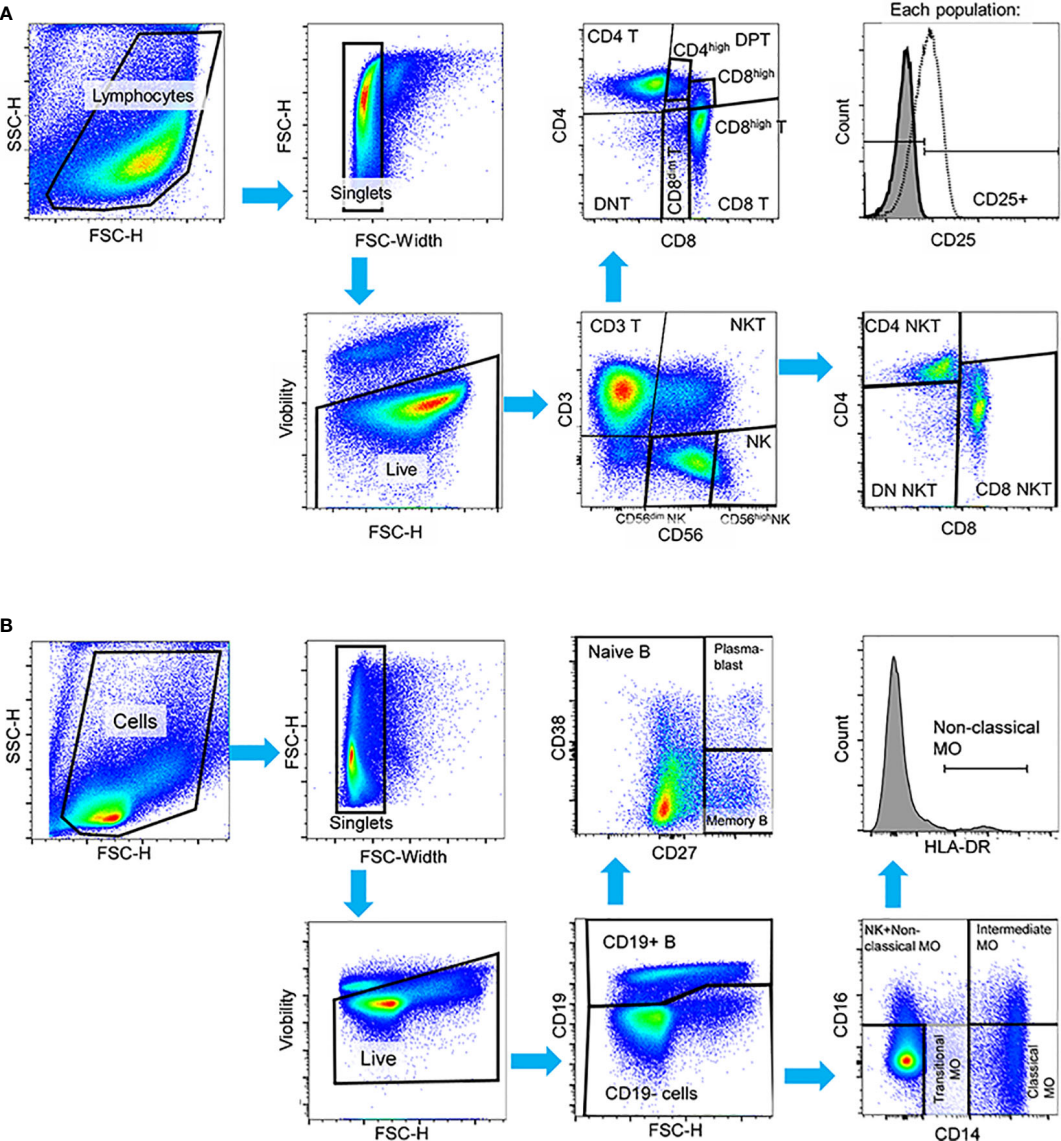
Manual gating strategy applied to PBMCs in both HC and SLE for the identification of **(A)** T-cell, NK-cell, NKT-cell subsets in the first panel, and **(B)** B-cell and monocyte subsets in the second panel in activated state. In both panels, cell debris, doublets and dead cells were excluded. **(A)** In the first panel, live cells were initially gated to CD3^+^ T-cells, CD56^dim^ NK-cells, CD56^high^ NK-cells and CD3^+^CD56^+^ NKT-cells. T-cells were further gated to CD4^+^ T-cells, CD8^+^ T-cells, CD4^+^CD8^+^ double positive T-cells (DPT) and CD4^-^CD8^-^ double negative (DNT). CD8^+^ T-cells were subdivided into CD8^dim^ and CD8^high^ T-cells and DPTs were categorized as CD4^high^ or CD8^high^ DPTs. NKTs were further gated to CD4^+^, CD8^+^ and CD4^-^CD8^-^ DN NKTs. The activation state was defined based on CD25 expression in this panel. **(B)** In the second panel, B-cells were identified by CD19 expression, and subsequent gating distinguished CD27^-^ naive B-cells, CD27^+^ memory B-cells, and CD27^++^CD38^++^ plasmablasts. CD19^-^ cells were further categorized into CD14^++^CD16^-^ classical monocytes, CD14^+^CD16^-^ transitional monocytes, and CD14^++^CD16^+^ intermediate monocytes. Non-classical monocytes were defined as CD14^+^CD16^++^HLA-DR^++^ cells. Representative pseudocolor plots and histograms are provided.

Flow cytometric data were analyzed in FlowJo v10.9.0. (Beckton Dickinson). Manual gating was used to determine main T-cell, NK-cell, NKT-cell, B-cell and monocyte populations within singlet living cells. Unstained cells were used as absolute negative control. The gating strategy for flow cytometry is shown in **Figure 1**. Lectin binding was calculated by subtracting the background median fluorescence intensity (MFI) values of the antibody-only samples from the MFI of all-lectin-plus-antibody samples. Main immune populations were exported, downsampled and concatenated using DownSample v3.3.1 plugin in FlowJo. High-dimensional data reduction and visualization were performed using t-distributed stochastic neighbor embedding (t-SNE) algorithm. Concatenated populations were also clustered into metaclusters by self-organizing-map based FlowSOM method (10). The number of metaclusters was determined as follows: every gated population from all the samples were concatenated separately and X-shift clustering was performed on the these concatenates of each population (49). Subsequently, the average X-shift cluster number was calculated, that was 5.44 (**Supplementary Table 5**). FlowSOM metaclusters were overlayed on the t-SNE plots using ClusterExplorer v.1.7.6. plugin.

### 2.8 Measurement of interferon concentrations by the LEGENDplex™ assay

The interferons were measured by the LEGENDplex™ assay. The LEGENDplex™ technology, a multiplex bead-based immunoassay (LEGENDplex™ Human Type 1/2/3 Interferon Panel (5-plex) with V-bottom Plate, Cat num.:740396, Biolegend) was used to determine the concentrations of the 5 interferons: (IFN-α2, IFN-β, IFN-λ1 (IL-29), IFN-λ2/3 (IL28a/b), IFN-γ) following to the instructions of the manufacturer. Briefly, 50 µL of undiluted plasma samples were incubated with the premixed capture beads and following washing, detection antibodies were added. Without washing, streptavidin-PE was added to each well directly. A standard curve was generated by the application of the commercial cytokine standards of the kit. Detection range was 51 ng/mL to 2.197 pg/mL. Samples were acquired on a Cytoflex S FACS (Beckman Coulter) using the APC and PE channels. Evaluation was performed in CytExpert 4.0 (Beckman Coultrer) and Microsoft Excel fitting the median values to the curve of the standards. Data are expressed as arithmetic mean of two replicate values in pg/mL. The IFN-β was not detectable, therefore, it is not included in the figure.

### 2.9 Statistics

Statistical analysis was performed using GraphPad Prism Version 8.4.2. For some data sets, normal distribution and lognormality could be excluded by Shapiro-Wilk, D’Agostino & Pearson and Kolmogorov-Smirnov normality and lognormality tests. For the rest of the data sets, distribution is unknown due to small sample size. In both cases, the assumption of normality was not satisfied for parametric tests. Therefore, uniformly nonparametric Wilcoxon matched-pairs signed rank test was used to compare data obtained in cell population of healthy controls and SLE patients. The significant differences were indicated as follows: * P ≤ 0.05, ** P ≤ 0.01, *** P ≤ 0.001.

## 3 Results

### 3.1 Manual gating and frequencies of main immune subsets in SLE

PBMCs obtained from SLE patients and healthy control (HC) individuals underwent analysis using two distinct panels: 1) the T-cell/NK-cell/NKT-cell panel and 2) the B-cell/monocyte panel. This analysis was performed shortly after thawing, referred to as “resting state”, and 72 hours after activation, referred to as “activated state”. The multiplex panel enabled us to analyze the main cell populations and subpopulations depicted in **Figure 1** alongside with the gating strategy of both panels.

**Figure 2:**
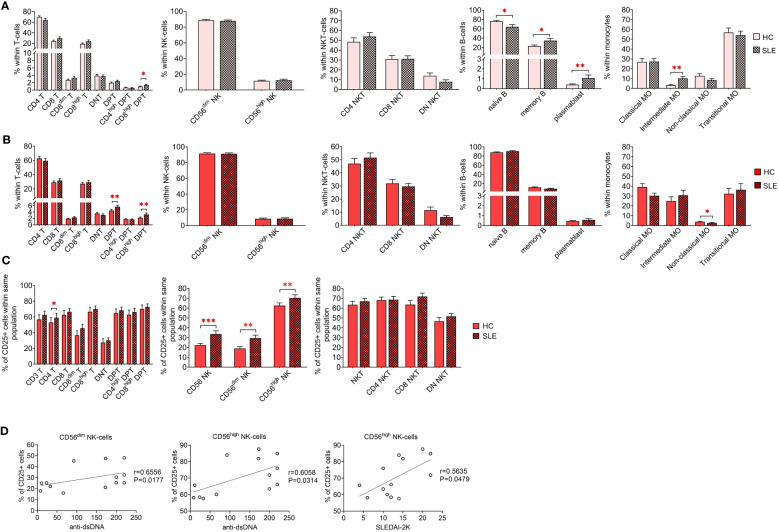
Frequencies of main immune subsets identified in PBMCs of heathy control (HC) individuals and SLE patients. Frequencies were measured in the **(A)** unstimulated resting state and **(B)** after 72-hour activation. T-cells, NK-cells and NKT-cells were stimulated with CytoStim™ and recombinant IL-2 (n = 13 for HC; n = 13 for SLE), while B-cells and monocytes received a cocktail of LPS and TLR9 agonist (n = 18 for HC, n = 18 for SLE). **(C)** For the activated cells of the first panel, the percentage of CD25^+^ cells within each population is shown (*P ≤ 0.05, **P ≤ 0.01, ***P ≤ 0.001). **(D)** Spearman correlation between the population frequencies of the SLE patients and their SLEDAI-2K scores or anti-double stranded DNA (anti-ds-DNA) antibody levels. Each point on scatter plot represents one individual subject, and the line represents the linear regression. Next to each plot, Spearman correlation coefficients (r) and associated P values.

Initially, a comparative analysis of population frequencies between HC and SLE was conducted under both resting and activated conditions. In the resting state, SLE PBMCs exhibited significantly higher frequencies of CD8^high^ DPTs, memory B-cells, plasmablasts, and intermediate monocytes, while naive B-cells were significantly lower compared to HC PBMCs (**Figure 2A**). **Figure 2B** illustrates the frequencies of the whole activated PBMC pool, revealing a significant increase in total DPTs, CD8^high^ DPTs, along with a significant decrease in non-classical monocytes in SLE. For the T-cells, NK-cells and NKT-cells, the positive fraction of the population for the activation marker CD25^+^ was defined in the whole PBMC, and the percentage of CD25^+^ CD4 T-cells, CD56^dim^ NK-cells CD56^high^ NK-cells was significantly elevated in SLE indicating increased activation (**Figure 2C**). Notably, a positive correlation was observed between the increased CD25 positivity of CD56^dim^ and CD56^high^ NK-cells and the anti-double stranded DNA antibody levels of the SLE patients. Additionally, a positive correlation was found between the CD25 positivity of CD56^high^ NK-cells and the SLEDAI-2K indices of the patients (**Figure 2D**).

Next, we examined the binding affinities of five distinct fluorescent dye-conjugated lectins (AAL, Gal-1, Gal-3, SNA and Siglec-1) to the previously defined immune subsets by comparing median fluorescence intensities (MFI) of each population. Subsequently, these MFI values, along with lineage-specific CD marker expression data, were utilized to identify unique populations using FlowSOM. The frequencies of these identified populations will be compared between HCs and SLE patients. Finally, to assess the clinical relevance of these measurements, parameters significantly altered in SLE subjects were correlated with their SLEDAI-2K indices and anti-double stranded DNA values.

### 3.2 Lectin binding analysis of T-cell subsets

**Figure 3:**
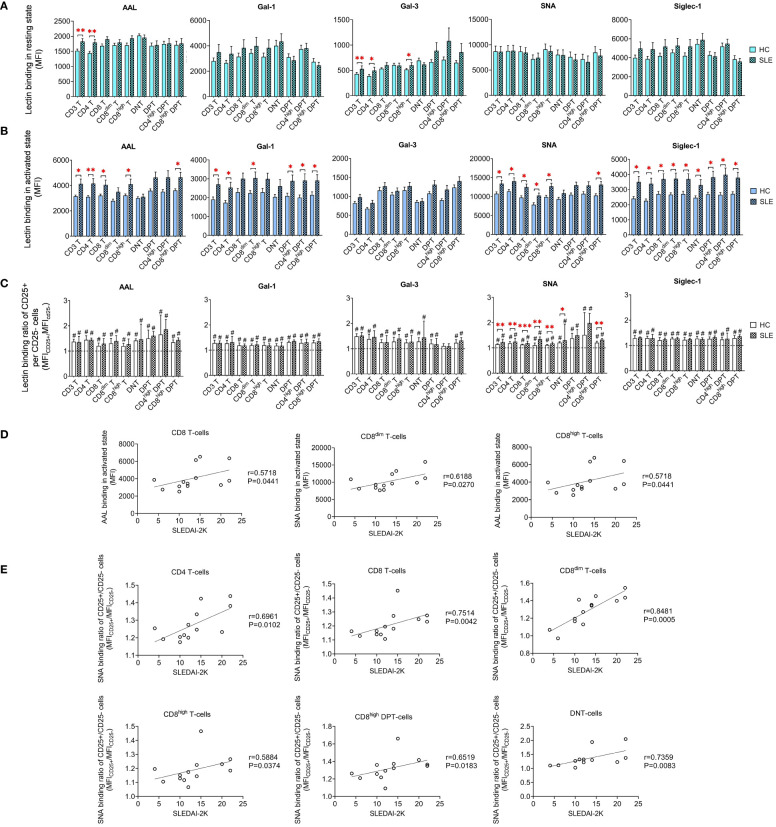
Binding capacity of five lectins (AAL, Gal-1, Gal-3, SNA and Siglec-1) to T-cell subsets in PBMCs of 13 SLE patients and 13 age- and sex-matched healthy control (HC) individuals analyzed by flow cytometry. The binding of fluorochrome-conjugated lectins was quantified as median fluorescence intensity (MFI) in the **(A)** unstimulated resting state and **(B)** after 72-hour activation. In the latter case, T-cells in the whole PBMC sample were stimulated with CytoStim™ and recombinant IL-2. **(C)** For the activated T-cells, lectin binding ratio was calculated between CD25^+^ and CD25^-^ cells within each T-cell subset (MFI_CD25+_/MFI_CD25-_). The symbol “#” marks significant difference (P ≤ 0.05) between CD25^+^ and CD25^-^ fractions, and asterisks between HC and SLE, * P ≤ 0.05, ** P ≤ 0.01, *** P ≤ 0.001. Data represents the mean + SEM (solid bars: HC; striped: SLE). **(D)** Spearman correlation between the lectin binding of activated T-cell subsets of SLE patients and their SLEDAI-2K indices. **(E)** Spearman correlation between the lectin binding ratio of activated CD25^+^ per CD25^-^ T-cell subsets of SLE patients and their SLEDAI-2K scores. Each point on scatter plot represents one subject, and the line represents the linear regression. Next to each plot, Spearman correlation coefficients (r) and associated P values.

Limited variations in lectin binding were noted between HC and SLE in resting T-cell subsets. Notably, there was a significant increase in the binding of AAL and Gal-3 binding in CD3 T-cells and CD4 T-cells. Additionally, SLE CD8^high^ T-cells exhibited heightened Gal-3 binding compared to their HC counterparts (**Figure 3A**). However, upon activation, distinct differences emerged in the lectin binding profile of HC and SLE T-cells (**Figure 3B**). Total CD3 T-cells including CD4 T-cells displayed significantly increased AAL, Gal-1, SNA and Siglec-1 binding in SLE. CD8 T-cells including their major subset CD8^high^ T-cells were characterized by significantly elevated AAL, SNA and Siglec-1 binding in SLE, whereas SLE CD8^dim^ T-cells also bound significantly higher amount of Gal-1, SNA and Siglec-1. DNT-cells only showed a notable increase in Siglec-1 binding in SLE. Conversely, within DPT-cells the CD4^high^ DPT-cell subpopulation demonstrated significantly elevated Gal-1 and Siglec-1 binding. SLE CD8^high^ DPT-cells were distinguished from HC counter population by significantly greater AAL, Gal-1, SNA and Siglec-1 binding as well. Furthermore, lectin binding ratio was calculated between CD25^+^ and CD25^-^ T-cell subsets shown in **Figure 3C** revealing significant upregulation in all lectin binding capacities in both HC and SLE T-cell subsets in **Figure 3C**, with the exception of Gal-3 in CD4^high^ DPTs. Notably, there were significant differences in the rate of upregulation between HC and SLE. CD3 T-cells, CD4 T-cells, CD8 T-cells and DNT-cell populations as well as CD8^dim^ T-cells, CD8^high^ T-cells and CD8^high^ DPT-cell subpopulations were characterized by significantly more robust SNA binding ratio in SLE than in HC (**Figure 3C**).

In CD8 T-cells, encompassing the major CD8^high^ T-cells subset, AAL binding in activated state positively correlated with SLEDAI-2K scores (**Figure 3D**). In activated CD8^dim^ T-cells, SNA binding demonstrated positive correlation with SLEDAI-2K scores (**Figure 3D**). The SNA binding ratio in activated total CD3 T-cells, including main subsets such as CD4 T-cells, CD8 T-cells and DNT-cells positively correlated with SLEDAI-2K. Similar associations were observed in activated subpopulations of CD8^dim^ T-cells, CD8^high^ T-cells and CD8^high^ DPT-cells (**Figure 3E**).

**Figure 4:**
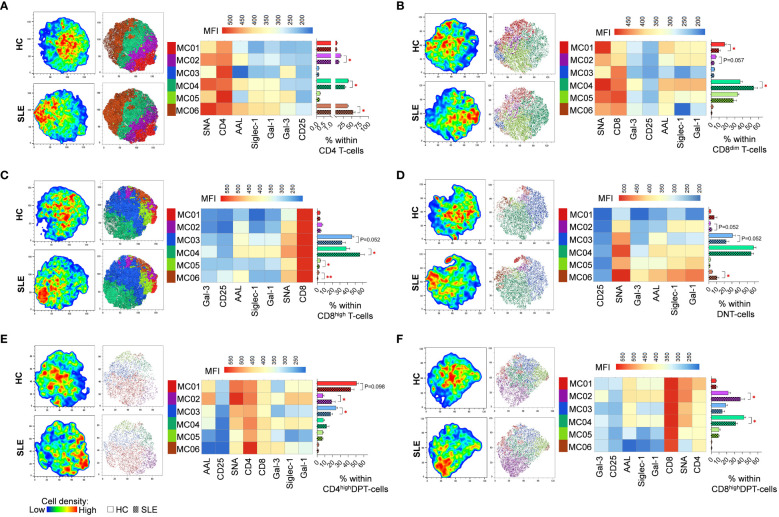
FlowSOM combined with t-SNE for the identification of metaclusters within manually gated activated T-cell subsets of 13 SLE patients and 13 age- and sex-matched HCs. **(A)** CD4 T-cell, **(B)** CD8^dim^ T-cell, **(C)** CD8^high^ T-cell, **(D)** DNT-cell, **(E)** CD4^high^ DPT-cell and **(F)** CD8^high^ DPT-cell populations of HCs and SLE patients were concatenated for this analysis. On the left t-SNE plots, colors vary according to cell abundance density, while on the right t-SNE plots color-coded FlowSOM metaclusters (MC01-MC06) are mapped. The median fluorescence intensity (MFI) profile of each FlowSOM metacluster is illustrated in a heatmap with rows representing each cluster and columns the markers of interest. Bar charts denote the frequency of each metacluster within the analyzed T-cell subset (solid bar: HC; striped bar: SLE). * p ≤ 0.05, ** p ≤ 0.01. Data represents the mean + SEM.

FlowSOM identified distinct metaclusters based on lectin binding properties of activated T-cell subsets. In CD4 T-cells, MC06 with the highest SNA binding and CD25 expression was significantly higher in SLE (54.0%) than in HC (37.3%). Conversely, MC02 (16.9% vs. 11.3%) and MC04 (39.6% vs. 29.1%) were significantly diminished in SLE (**Figure 4A**). In healthy donors’ CD8^dim^ T-cells, over a third of the cells (36.8%) belonged to MC04 exhibiting the strongest binding of all five lectins within the group and relatively high CD8 expression. In SLE, MC04 constituted 53.8% of CD8^dim^ T-cells resulting in a significant difference between HC and SLE (**Figure 4B**). The SNA^high^ MC01 with moderate binding of other lectins and lower CD8 expression was significantly lower in SLE (9.8%) compared to HCs (17.1%) (**Figure 4B**). Similarly, in SLE CD8^high^ T-cells, the predominant metacluster with outstanding lectin binding was MC04 (56.2%), which was significantly smaller in HCs (38.2%), whereas there was a notable but statistically not significant difference (P=0.052) in the frequency of less glycosylated MC03 between HC and SLE (45.8% vs. 32.7%). Additionally, smaller metaclusters MC05 (4.0% vs. 2.6%) and MC06 (2.1% vs. 0.8%) were significantly higher in HC than in SLE (**Figure 4C**). In DNT-cells, MC06 with exceptionally high Gal-1 binding was significantly increased in SLE (10.6%) compared to HC (4.1%) and MC03 was almost significantly lower (P=0.052) in SLE (22.5%) than in HC (39.0%) (**Figure 4D**). Moving to CD4^high^ DPT-cells, the frequency of MC02, the metacluster with maximal AAL, Gal-1, SNA and Siglec-1 binding across the group, more than doubled in SLE (18.7%) compared to HC (7.5%). Other metaclusters with less pronounced glycosylation such as MC01 and MC03 were underrepresented in SLE (43.3% and 17.4%) compared to HC (50.9% and 24.4%), with the difference in MC03 being statistically significant (**Figure 4E**). Finally, in CD8^high^ DPT-cells, the proportion of cells in MC02 was significantly heightened in SLE (37.6%) compared to the percentage of MC02 cells in HC (21.9%) and MC04, characterized by relatively lower lectin binding compared to MC02, was significantly less prevalent in SLE (31.6%) than in HC (42.3%) (**Figure 4F**).

### 3.3 Lectin binding analysis of NK-cell subsets

**Figure 5:**
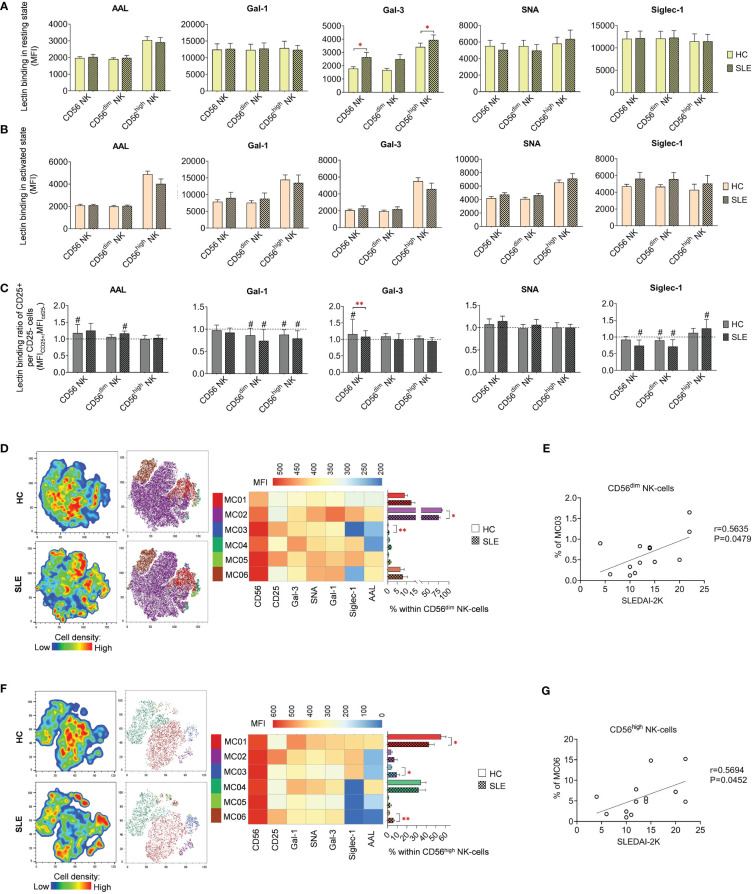
Lectin binding properties and FlowSOM clustering analysis of manually gated NK-cell subsets of 13 SLE patients and 13 age- and sex-matched HCs. The binding of fluorochrome-conjugated lectins was quantified as median fluorescence intensity (MFI) in the **(A)** unstimulated resting state and **(B)** after 72-hour activation. In the latter case, NK-cells in the whole PBMC sample were stimulated with CytoStim™ and recombinant IL-2. **(C)** For the activated NK-cells, lectin binding ratio was calculated between CD25^+^ and CD25^-^ cells within each NK-cell subset (MFI_CD25+_/MFI_CD25-_). The symbol “#” marks significant difference (P ≤ 0.05) between CD25^+^ and CD25^-^ fractions. For the clustering analysis, activated **(D)** CD56^dim^ NK-cell and **(F)** CD56^high^ NK-cell populations of HCs and SLE patients were concatenated and FlowSOM was combined with t-SNE visualization. On the left t-SNE plots, varying colors correspond to relative cell density, while on the right t-SNE plots FlowSOM metaclusters (MC01-MC06) are shown with different colors. Heatmap is generated from the MFI profile of FlowSOM metaclusters with rows representing each cluster and columns the markers of interest. Bar graphs denote the percentage of metaclusters within the analyzed NK-cell subset. Data are presented as mean + SEM (solid bars: HC; striped bars: SLE). *P ≤ 0.05, **P ≤ 0.01. Spearman correlation between the metacluster frequencies of CD56^dim^ **(E)** and CD56^high^ **(G)** NK-cells of SLE patients and their SLEDAI-2K scores. Each point on scatter plot represents one subject, and the line represents the linear regression. Next to each plot, Spearman correlation coefficients (r) and associated p values.

In quiescent state, significant elevations were observed in Gal-3 binding in total CD56 NK-cells and CD56^high^ NK-cells (**Figure 5A**). However, no noteworthy changes were detected in lectin binding in activated state (**Figure 5B**). In contrast to T-cell subsets, the activation hallmarked in CD25 positivity was not uniformly linked to elevated lectin binding. Notably, only in a few cases exhibited CD25^+^ NK-cell subsets significant increase in lectin binding. Specifically, AAL and Gal-3 showed a significant increase in total HC CD56 NK-cells, while AAL in CD56^dim^ NK-cells and Siglec-1 in CD56^high^ NK-cells demonstrated significant elevation in SLE (**Figure 5C**). The Gal-3 binding ratio between CD25^+^ and CD25^-^ total CD56 NK-cells was significantly lower in SLE compared to HC (**Figure 5C**).

Although the lectin binding pattern of activated NK-cell subsets did not differ significantly in SLE, FlowSOM unveiled alterations in metacluster frequencies in SLE. In CD56^dim^ NK-cells, the predominant metacluster MC02 (82.8% in HC) with prominent Gal-1, SNA and Siglec-1 binding was significantly decreased in SLE (75.5%) and second largest metacluster MC01 (9.0% in HC), distinguished by relatively weak lectin binding, was elevated in SLE (12.7%) (**Figure 5D**). The small metacluster MC03, characterized by strong CD25 expression but negligible amount of AAL and Siglec-1 binding, was significantly overrepresented in SLE (0.64%) compared to HC (0.2%). The percentage of this metacluster in SLE CD56^dim^ NK-cells positively correlated with the SLEDAI-2K indices of the patients (**Figure 5E**). In HC CD56^high^ NK-cells, more than half of the cells (55.4%) belonged to MC01, with maximal lectin binding within the subset significantly underrepresented in SLE (42.9%). Consequently, there was a significant increase in other metaclusters, namely MC03 (9.3% in SLE vs. 3.7% in HC) and MC06 (6.0% in SLE vs. % in HC) (**Figure 5F**). MC03 had diminished Gal-1, SNA, Gal-3 and especially AAL but higher Siglec-1 binding compared to MC01. MC06 showed extremely low AAL and Siglec-1 binding but strong CD25 expression. Interestingly, the proportion of MC06 obtained positive correlation with SLEDAI-2K scores of the SLE patients (**Figure 5G**).

### 3.4 Lectin binding analysis of NKT-cell subsets

**Figure 6:**
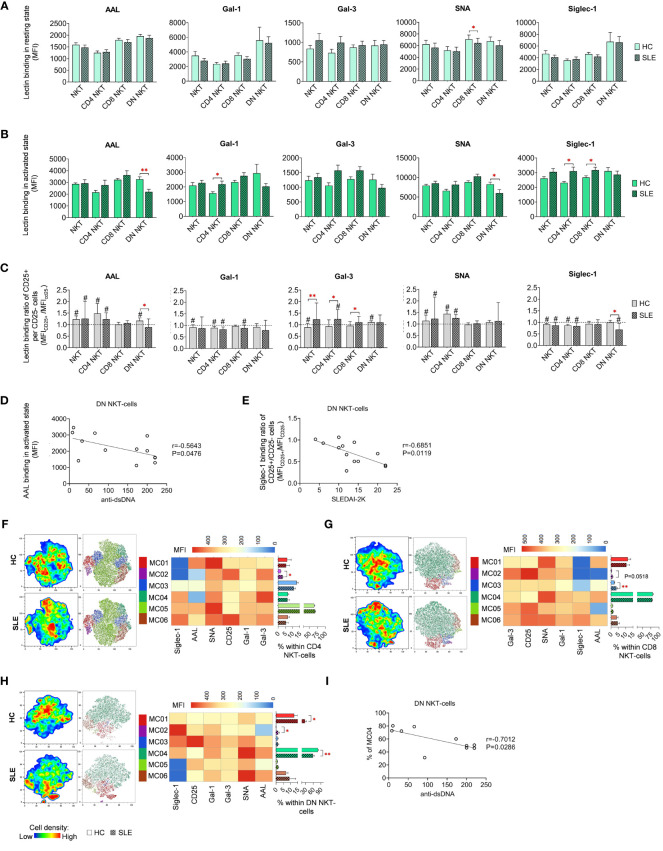
Lectin binding profile and FlowSOM clustering analysis of manually gated NKT-cell subsets of 13 SLE patients and 13 age- and sex-matched HCs. The binding of five fluorochrome-conjugated lectins was measured as median fluorescence intensity (MFI) in the **(A)** unstimulated resting state and **(B)** after 72-hour activation. In the latter case, NKT-cells in the pool of PBMCs were stimulated with CytoStim™ and recombinant IL-2. **(C)** For the activated NKT-cells, the lectin binding ratio was calculated between CD25^+^ and CD25^-^ cells within each NKT-cell subset (MFI_CD25+_/MFI_CD25-_). The symbol “#” marks significant difference (P ≤ 0.05) between CD25^+^ and CD25^-^ fractions, and asterisks denote significance between HC and SLE. Spearman correlation was performed between the **(D)** AAL binding properties or **(E)** Siglec-1 CD25^+^ per CD25^-^ binding ratio of activated DN NKT-cells of SLE patients and their SLEDAI-2K or anti-ds-DNA antibody values. Each point on scatter plot represents one subject, with the line indicating linear regression. Next to each plot, Spearman correlation coefficients (r) and associated P values. For clustering analysis, activated **(F)** CD4 NKT-cell, **(G)** CD8 NKT-cell and **(H)** DN NKT-cell populations from both HCs and SLE patients were concatenated, and FlowSOM was combined with t-SNE visualization. On the left t-SNE plots, color gradients from dark blue to dark red indicates increasing cell density, whereas on the right t-SNE plots color-coded FlowSOM metaclusters (MC01-MC06) are displayed. The MFI profile of each FlowSOM metacluster is visualized in a heatmap. Bar charts denote the frequency of each metacluster within the analyzed NK-cell subset. **(I)** Spearman correlation between the MC04 metacluster frequency of DN NKT-cells of SLE patients and their anti-ds-DNA antibody values. Each point on scatter plot represents one subject, and the line represents the linear regression. Adjacent to the plot, Spearman correlation coefficient (r) and associated P value. Data are presented as mean + SEM (solid bars: HC; striped bars: SLE). *p ≤ 0.05, **p ≤ 0.01.

In resting state, SLE CD8 NKT-cells bound significantly less SNA than their counterparts from healthy donors (**Figure 6A**). In activated state, both CD4 and CD8 NKT-cells in SLE exhibited significantly higher Siglec-1 binding and SLE CD4 NKT-cells also bound significantly more Gal-1 than HC CD4 NKT-cells (**Figure 6B**). On the other hand, DN NKT-cells in SLE showed significantly decreased AAL and SNA binding (**Figure 6B**). Importantly, a negative correlation was observed between the reduced AAL binding in SLE DN NKT-cells and the elevated anti-double-stranded DNA antibody levels in the same patients (**Figure 6D**). Furthermore, there was no clear trend observed in the lectin binding ratios of CD25^+^ and CD25^-^ NKT-cell subsets, although significant differences between HC and SLE are noted (**Figure 6C**). In the CD25^+^/CD25^-^ AAL lectin binding ratio, there was a significant reduction in SLE DN NKTs. Regarding Gal-3, total SLE NKT-cells including CD4 NKT- and CD8 NKT-cell subsets, lectin binding ratio was significantly elevated compared to that of HCs. In SLE, there was a significant decline in Siglec-1 binding ratio in DN NKT-cells (**Figure 6C**). This decreased Siglec-1 binding ratio of CD25^+^ and CD25^-^ DN NKT-cells in SLE patients negatively correlated with the patients’ SLEDAI-2K indices (**Figure 6E**).

The unsupervised FlowSOM approach identified phenotypically distinct subpopulations in NKT-cell subsets revealing particularly notable differences in AAL and Siglec-1 binding. In CD4 NKT-cells, small metacluster MC02, characterized by minimal AAL and Siglec-1 binding alongside high CD25 expression, was significantly expanded in SLE (3.1%) compared to HCs (1.9%) (**Figure 6F**). Similarly, in CD8 NKT-cells, MC02 with negligible AAL and Siglec-1 binding, and MC03 with intermediate AAL and low Siglec-1 binding, were enriched in SLE (1.1% and 3.7%, respectively) compared to HC (0.4% and 2.3%, respectively), with the latter difference being statistically significant (**Figure 6G**). In the case of DN NKT-cells, MC01 (12.6% in HC) exhibiting moderate lectin binding properties, more than doubled in SLE (27.7%) (**Figure 6H**). The second metacluster, MC02, characterized by high Siglec-1 and low AAL binding, was nearly absent in HC (0.1%), albeit its ratio was increased in SLE (1.5%). Lastly, the largest metacluster MC04, featuring high AAL, Gal-1, SNA and Siglec-1 binding, was significantly underrepresented in SLE (58.8%) in comparison to HC (78.5%). Consistent with earlier findings, the prevalence of MC04 in DN NKT-cells of SLE patients showed a negative correlation with the patients’ anti-ds DNA antibody levels (**Figure 6I**).

### 3.5 Lectin binding analysis of B-cell subsets

**Figure 7:**
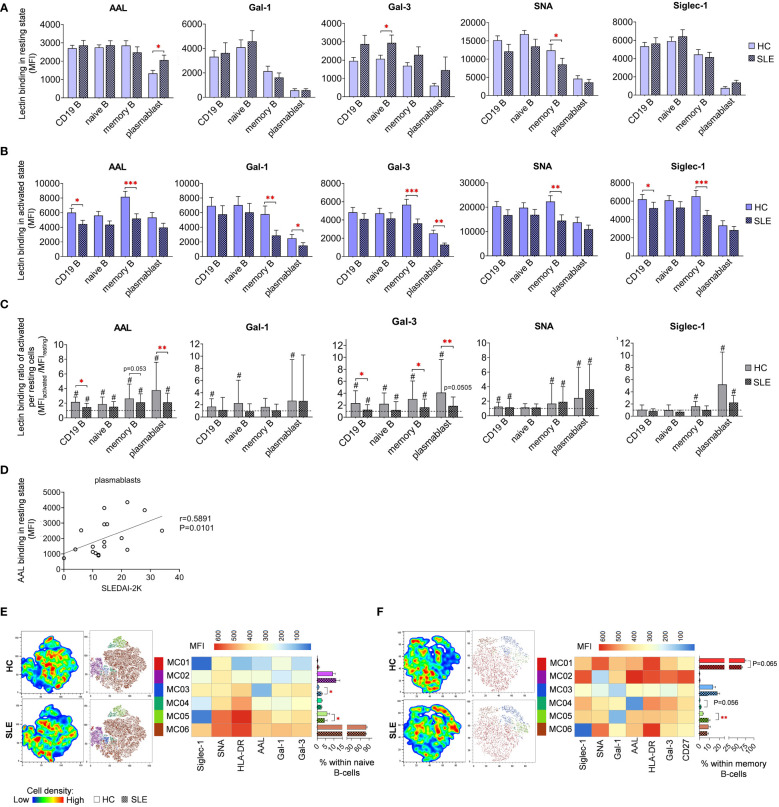
Lectin binding properties and FlowSOM clustering analysis of manually gated B-cell subsets of 18 SLE patients and 18 age- and sex-matched HCs. The binding of fluorochrome-conjugated lectins was quantified as median fluorescence intensity (MFI) in the **(A)** unstimulated resting state and **(B)** after 72-hour activation. In the latter case, B-cells in the whole PBMC sample were stimulated with LPS and TLR9 agonist. **(C)** The lectin binding ratio between resting and activated samples was calculated as MFI_activated_/MFI_resting_ within each B-cell subset, with # indicating significant differences (p ≤ 0.05). **(D)** Spearman correlation between the AAL lectin binding properties of resting plasmablasts of SLE patients and their SLEDAI-2K indices. Each point on scatter plot represents an individual subject, and the line represents the linear regression. Next to the plot, Spearman correlation coefficient (r) and the associated P value. For the clustering analysis, activated **(E)** naive B-cell and **(F)** memory B-cell populations of HCs and SLE patients were concatenated and FlowSOM was combined with t-SNE visualization. On the left t-SNE plots, varying colors correspond to relative cell density, while on the right t-SNE plots FlowSOM metaclusters (MC01-MC06) are overlayed with different colors. The MFI profile of each FlowSOM metacluster is illustrated in a heatmap where rows represent individual clusters and columns represent markers of interest. Bar graphs denote the percentage of metaclusters within the analyzed B-cell subset. Data are presented as mean + SEM (solid bars: HC; striped bars: SLE). *p ≤ 0.05, **p ≤ 0.01, ***p ≤ 0.001.

In resting state, fucosylation of plasmablasts in SLE as measured by AAL binding exhibited substantial increase compared to HC (**Figure 7A**). Intriguingly, positive correlation was discerned between the AAL binding of plasmablasts and the SLEDAI-2K scores of SLE patients (**Figure 7D**). Additional distinctions observed in SLE in resting state include elevated Gal-3 binding in naive B-cells and reduced SNA binding in memory B-cells (**Figure 7A**). In activated state, total CD19 B-cells showed significantly decreased AAL and Siglec-1 binding (**Figure 7B**). Notably, there was a clear trend of markedly lower binding of the five lectins in memory B-cells in SLE. In comparison to HC, SLE plasmablasts also bound significantly less Gal-1 and Gal-3. Changes in lectin binding upon activation are depicted in **Figure 7C**, expressed as binding ratios between the two conditions across the same dataset. Total CD19 B-cells, memory B-cells and plasmablasts performed significantly lower AAL and Gal-3 binding ratios in SLE, except AAL in memory B-cells (P=0.053).

FlowSOM was applied to discover phenotypically distinct subsets in naive and memory B-cells based on their lectin binding profile, HLA-DR activation marker expression, and, in case of memory B-cells, CD27 expression. Unfortunately, the constrained cell counts of plasmablasts precluded subsequent clustering. In naive B-cells, approximately 80% (80.6% in HC and 78.3% in SLE) of the cells were classified into MC06 characterized by robust lectin binding and strong HLA-DR expression (**Figure 7E**). The small metacluster MC03, with lowest AAL binding and moderate binding of other lectins, was significantly expanded in SLE (2.6%) compared to HC (1.0%). Additionally, the distribution of MC05, with outstanding AAL and SNA binding, and HLA-DR expression but relatively low Siglec-1 binding, was significantly reduced in SLE (4.4%) compared to HC (6.0%) naive B-cells (**Figure 7E**). A majority of memory B-cells belonged to MC01 distinguished by high HLA-DR expression, prominent Gal-1 and SNA binding (**Figure 7F**). The proportion of this metacluster in HC exceeded that in SLE by 10% (62.4% vs. 72.3%). Consequently, other metaclusters in the memory B-cell subset were enriched in SLE. Accordingly, MC03 displaying a poor lectin binding pattern, was increased in SLE by almost 5% (18.1% vs. 13.7%). Furthermore, the proportion of MC04 with negligible AAL binding was more than doubled in SLE (1.7% vs. 0.7% in HC), whereas MC05 featuring the lowest Gal-1 binding and intermediate HLA-DR expression, was significantly more prevalent in SLE (9.2% vs. 3.8% in HC) (**Figure 7F**).

Since metaclusters of the B-cell subsets with significantly different abundance in SLE showed altered HLA-DR expression in many cases, the MFI values of HLA-DR expression is presented in both resting (**Supplementary Figure 1A**) and activated states (**Supplementary Figure 1B**). Interestingly, all B-cell subsets expressed tendentiously less HLA-DR on the cell surface in SLE. Notably, in case of memory B-cells in activated state, the difference reached statistical significance (**Supplementary Figure 1B**). Stimulation by LPS and TLR9 agonist induced significant upregulation of HLA-DR in both HC and SLE B-cell subsets, although the extent of upregulation was significantly greater in SLE memory B-cells and plasmablasts (**Supplementary Figure 1C**).

### 3.6 Lectin binding analysis of monocyte subsets

**Figure 8:**
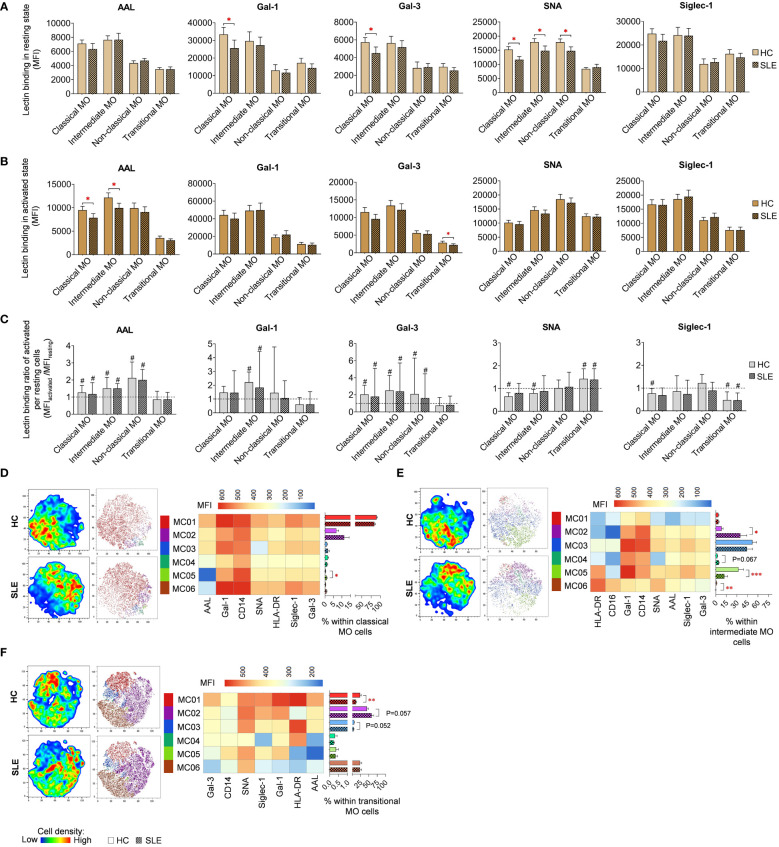
Lectin binding profile and FlowSOM clustering analysis were conducted on manually gated monocyte (MO) cell subsets of 18 SLE patients and 18 age- and sex-matched HCs. The binding of five fluorochrome-conjugated lectins was measured as median fluorescence intensity (MFI) in **(A)** the unstimulated resting state and **(B)** after 72-hour activation. In the latter case, monocytes in the PBMC pool were stimulated with a cocktail of LPS and TLR9 agonist. **(C)** The lectin binding ratio was calculated across all subsets between samples of unstimulated resting and activated state (MFI_activated_/MFI_resting_). The symbol “#” denotes significant difference (P ≤ 0.05) between resting and activated states, while asterisks indicate significance between HC and SLE (P ≤ 0.05 *, P ≤ 0.01 **, P ≤ 0.001***). For clustering analysis, activated **(D)** classical monocytes, **(E)** intermediate monocytes and **(F)** transitional monocyte populations of HCs and SLE patients were concatenated, and FlowSOM was combined with t-SNE visualization. On the left t-SNE plots, color gradients from dark blue to dark red indicate increasing cell density, whereas on the right t-SNE plots, color-coded FlowSOM metaclusters (MC01-MC06) are mapped. The MFI profile of each FlowSOM metacluster is shown in a heatmap. Bar charts denote the frequency of each metacluster within the analyzed monocyte subset. Data are presented as mean + SEM (solid bars: HC; striped bars: SLE). *P ≤ 0.05, **P ≤ 0.01, ***P ≤ 0.001.

In resting classical monocytes (MO) of SLE patients, there was a significant reduction in Gal-1, Gal-3 and SNA binding (**Figure 8A**). Moreover, SNA demonstrated significantly lower binding to intermediate and non-classical monocytes in SLE. In the activated state, the level of AAL binding was significantly declined in classical and intermediate monocytes in SLE, and transitional monocytes in SLE bound significantly lower amount of Gal-3 (**Figure 8B**). Activation by LPS and TLR9 agonist triggered significant changes in lectin binding of both HC and SLE monocyte subsets, and there was no significant difference between HC and SLE in the lectin binding ratio between the activated and resting state (**Figure 8C**).

LPS and TLR9 agonist stimulated classical, intermediate and transitional monocytes subsets were subjected to clustering utilizing FlowSOM and were organized into distinct subtypes. Due to insufficient cell count in the non-classical monocytes, clustering analysis was not conducted for this population. The majority of classical monocytes (89.6% in HC) were categorized by FlowSOM into MC01, which had the highest lectin binding and also strong CD14, and HLA-DR expression (**Figure 8D**). In SLE, the prevalence of MC01 decreased by approximately 6% (83.7%), leading to the elevation of other metaclusters, namely MC02 and MC03, with less pronounced lectin binding and HLA-DR expression, were elevated in SLE (11.4%; 1.7%) compared to HC (6.8%; 1.2%). Additionally, MC05, with a lower lectin binding profile compared to MC01 and the smallest AAL binding across all metaclusters in this group, was significantly more abundant in SLE (1%) than in HC (0.3%). More pronounced changes were detected in intermediate monocytes (**Figure 8E**), where MC02 exhibiting a CD14^high^ CD16^low^ HLA-DR^low^ Gal-1^intermediate^ phenotype, was remarkably expanded in SLE compared to HC (35.0% vs 8.3%). Conversely, MC05, with CD14^intermediate^ CD16^intermediate^ HLA-DR^high^ Gal-1^high^ characteristics, was significantly decreased in SLE (12.3% vs 32.4%). MC06, displaying a CD14^low^ CD16^high^ HLA-DR^high^ Gal-1^low^ SNA^high^ phenotype, was significantly less abundant in SLE than in HC (0.2% vs. 1.3%) (**Figure 8E**). In transitional monocytes, MC01, characterized by maximal AAL, Gal-1, Gal-3 and Siglec-1 binding within the group, was significantly underrepresented in SLE (12.2%) in comparison to HC (24.2%), while MC02, demonstrating relatively weaker binding of the aforementioned lectins and low HLA-DR expression, comprised 44.8% of transitional MO cells in HC and 58.7% of the cells in SLE (58.7%) (**Figure 8F**).

For classical and intermediate MOs, significantly lower HLA-DR expression was detected in SLE in both conditions (**Supplementary Figures 1D, E**). Activated transitional monocytes also showed decreased HLA-DR expression in SLE compared to HC (**Supplementary Figure 1E**). In contrast, resting non-classical monocytes showed elevated HLA-DR expression (**Supplementary Figure 1D**). The expression ratio calculated between activated and resting state was significantly lower in SLE classical and intermediate monocytes than in their HC counterparts (**Supplementary Figure 1F**). Altered CD14 was only observed in resting classical monocytes, with a significant reduction in SLE (**Supplementary Figure 1D**). Upon activation, CD14 expression was significantly upregulated in classical, intermediate, and non-classical monocytes in both HC and SLE, although no difference was found in the rate of upregulation between the two subject groups (**Supplementary Figure 1F**). CD16 expression was significantly reduced in resting intermediate monocytes and in resting and activated non-classical monocytes in SLE compared to HC (**Supplementary Figures 1D, E**). Finally, classical monocytes in SLE demonstrated a significantly lower CD16 expression ratio between the activated and resting states (**Supplementary Figure 1F**).

### 3.7 Measurement of interferon concentrations in the plasma samples

Most of the research on SLE and type I interferons focused on IFN-α and IFN-β. Indeed, the differentiation and expansion of the pathogenic T-cell subsets are linked with the release of type I interferons (IFN−I), (2, 3) fundamental contributors to SLE pathogenesis. Another cells, plasmacytoid dendritic cells were identified as the main source of IFN-α and macrophages and fibroblasts the major source of IFN-β. Recently, studies highlighted the role for IFN-κ, mainly expressed in keratinocytes in response to exposure to UV light. The major challenge is that all types of the source cells are not there in PBMC samples, or their number is insufficient for culturing and measuring their effect. The tissue resident IFN-I producers could be identified *in situ* in the inflamed tissues. However, in our study these factors can be investigated from the stored plasma samples of the patients and HCs. The stored plasma samples from the HC and SLE patient cohort were analyzed by the LEGENDplex™ Human Type 1/2/3 Interferon Panel (BioLegend). Although, the average IFN concentrations were higher in the SLE group but there was no significant difference observed in IFN-α2, IFN-γ, IFN-λ1 and IFN-λ2 between HC and SLE (**Supplementary Figure 2**). The authors suppose that higher number of subjects could provide significant increase of IFNs in the plasma of SLE patients.

## 4 Discussion

### 4.1 T-cells

As demonstrated by us and others, stimulation prompts an increase in cell surface glycosylation, particularly enhancing glycan complexity in T-cells manifested in increased lectin binding (34, 50, 51). In the current work, the activation-induced elevation of alpa-2,6-sialylation was significantly more robust in SLE CD4 T-cells, CD8 T-cells, including CD8^dim^ and CD8^high^ T-cells, DNT-cells along with CD8^high^ DPT-cells than in HC counterparts. This was reflected in significantly higher SNA binding ratio between CD25^+^ and CD25^-^ fractions of these T-cell subsets in SLE. Consequently, activated SLE T-cell subsets bound significantly more SNA than HC T-cell subsets. These findings align with our previous results, which indicated a slight elevation in SNA binding in activated SLE T-cells, however, that difference did not reach statistical significance. This previously reported heightened alpha-2,6 sialylation of SLE T-cells compared to HC T-cells upon activation was further confirmed by a significant increase in the mRNA ratios of ST6 beta-galactoseamide alpha-2,6-sialyltranferase 1 and neuraminidase 1 (ST6GAL1/NEU1) in activated SLE T-cells responsible for adding and removal of sialic acids on glycans. (34) In contrast, others compared the sialylation of freshly isolated unstimulated CD4 T-cells from SLE patients and HCs, finding no notable difference in SNA binding (52). Interestingly, the FlowSOM clustering algorithm revealed the expansion of metaclusters with extreme SNA binding in SLE, emerging as the predominant metaclusters in SLE CD4 T-cells, CD8^dim^ T-cells, CD8^high^ T-cells and in CD8^high^ DPT-cells. These metaclusters were also activated expressing intermediate or high CD25 compared to other metaclusters within the same subset. Despite the increased prevalence of these CD25^medium^/CD25^high^ metaclusters in SLE, the overall CD25 positivity in SLE upon activation was only slightly increased in each T-cell subset, reaching statistical significance in the case of CD4 T-cells. It is important to note that hyperactivation is a well-established feature of SLE T-cells, attributed to both increased sensitivity of the TCR and enhanced downstream signaling (53). Interestingly, in murine models St6gal1 gene expression was found to be downregulated in both CD4 and CD8 T-cells following activation (29). Therefore, the connection between T-cell activation and alpha-2,6-sialylation remains inconclusive and requires further clarification in future investigations. On the flip side, T-cell activation was linked with core fucosylation of TCR catalyzed by fucosyltransferase 8 (FUT8). Intriguingly, hyperactivation of this enzyme has been documented in SLE CD4 T-cells (14). In our experiments, increase of core fucosylation was another hallmark of SLE T-cell subsets. The expanded metaclusters in SLE T-cells subsets mentioned earlier were also characterized by remarkable core fucosylation indicated by strong AAL binding. AAL binding was significantly increased in bulk activated populations of CD4 T-cells, CD8 T-cells including CD8^high^ subtype and CD8^high^ DPT cells from SLE. Most importantly, many of these altered parameters in alpha-2,6-sialylation and core fucosylation demonstrated a correlation with disease activity. The ratio of SNA binding in activated (CD25^+^) versus non-activated (CD25^-^) fraction of CD4 T-cells, CD8 T-cells, including CD8^dim^ and CD8^high^ T-cells, DNT-cells along with CD8^high^ DPT-cells T-cells positively correlated with SLEDAI-2K indices of the patients. Additionally, the SNA binding of activated CD8^dim^ T-cells and AAL binding of activated CD8 T-cells including CD8^high^ T-cells positively correlated with SLEDAI-2K indices of SLE patients. Our results support previous findings that the expression of Siglec-1-ligands on T-cells correlates with the activity of nephritis in a murine lupus model (54). Siglec-1 expression on monocytes is a Type-I interferon-inducible process, and its intensity correlates with serum soluble Siglec-1 levels and the presence of nephritis in SLE patients (35). The metaclusters significantly enriched in our SLE patients consistently show the highest level of Siglec-1-binding in all T-cell subsets examined. However, the effect of Siglec-1-binding on T-cell function needs to be clarified.

### 4.2 NK-cells

NK cells can be divided into cytolytic CD56^dim^ and immunomodulatory, cytokine producing CD56^high^ subsets (55). In SLE, the ratio of CD25^+^ cells was significantly elevated in total NK-cells as well as in CD56^dim^ and CD56^high^ subgroups in SLE. The percentage of CD25^+^ CD56^dim^ and CD56^high^ NK-cells of SLE patients positively correlated with anti-ds-DNA antibody levels, and the same value in CD56^high^ NKs showed positive correlation with SLEDAI-2K indices too. The heightened CD25-positivity in SLE can be partially explained by the dynamics of subsets with varying CD25 expression levels explored by FlowSOM. Specifically, majority of the stimulated CD56^dim^ and CD56^high^ NK-cells were categorized as CD25^low^, Gal-1^high^, Siglec-1^high^ SNA^high^ cells. In SLE, these metaclusters were significantly reduced in both cell types in SLE. Conversely, small, less glycosylated metaclusters with CD25^high^ AAL^low^ Siglec-1^low^ SNA^med^ phenotype (MC03 in CD56^dim^ and MC06 in CD56^high^ NKs) were significantly overrepresented in SLE. Enhanced activated phenotype in SLE NK-cells indicated by increased CD69 expression was also observed by other investigators in both CD56^dim^ and in CD56^bright^ subsets in association with disease severity (56, 57). Interestingly, within normal NK-cells high CD25 expression marked phenotypically different subset, since IL-15 primed, IL-2 restimulated CD25^bright^ NK-cells had higher pSTAT5 phosphorylation, proliferation rate (Ki67-positivity) and mitochondrial respiration than CD25^dim^ or CD25- counterparts (58). The authors concluded that this phenotype (CD25^bright^ Ki67^+^) might be beneficial in cancer, although in the context of autoimmunity, it could potentially drive pathogenic processes. Intriguingly, in SLE, the percentage of proliferating Ki67+ NK-cells positively correlated with SLEDAI-2K scores, the percentage of plasma cells and CD11c^+^ B-cells (59). In our experiments, the expanded metaclusters in SLE with an activated CD25^high^ phenotype, specifically CD56^dim^ MC03 and CD56^high^ MC06, also exhibited a positive correlation with SLEDAI-2K indices. Whether these metaclusters are characterized by high proliferation rate and altered metabolic activity must be addressed in the future. In this study, these subsets were defined by low Siglec-1 binding, moderate alpha-2,6-sialylation and low core fucosylation. Sialylation can impact the function of both activating (e.g. 2B4) and inhibitory receptors (Siglec-7, Siglec-9) on NK-cells (55, 60). Unfortunately, there is no information available on fucosylation of NK-cell specific molecules. The exact proteins with altered sialylation and fucosylation in MC03 and MC06 metaclusters must be elucidated, although the very low binding of the respective lectins could serve as sufficient biomarkers to identify these subsets.

### 4.3 NKT-cells

Here, we gated CD3^+^CD56^+^ T-cells, which phenotype can be acquired by conventional CD8^+^ T-cells alongside unconventional mucosal-associated invariant T-cells (MAIT), γδ-T-cells and NKT-cells (61). NKT cells can be further classified as CD1d-dependent type I (classical, termed also invariant NKTs – iNKTs), type II (non-classical) and CD1d-independent NKT-like cells (62). Based on CD4 and CD8 expression, NKTs can be subdivided into CD4^+^, CD8^+^ and DN subsets (63). In this study, the most striking differences among HC and SLE were observed in the DN NKT subset. Following stimulation, this subset exhibited a less pronounced increase in AAL binding in SLE compared to HC. The diminished AAL binding of activated DN NKTs obtained negative correlation with anti-dsDNA antibodies. Similarly, the Siglec-1 binding ratio of CD25^+^/CD25^-^ cells was significantly smaller in SLE than in HC and this ratio negatively correlated with SLEDAI-2K indices. Correspondingly, the largest DN NKT metacluster, MC04, characterized by high fucosylation, α-2,6-sialylation and strong Siglec-1 binding was significantly decreased in SLE, and the frequency of this metacluster negatively correlated with anti-dsDNA levels. These findings suggest that stimulation-induced changes in glycosylation of DN NKTs have defects in SLE in correlation with disease activity. Consistently, SLE iNKTs exerted functional defects in response to stimulation compared to iNKTs from HCs. The combination of IL-15 and α-galactosyl ceramide triggered reduced expansion and perforin expression of iNKTs in SLE compared to controls. Moreover, in response to the same stimulation, SLE iNKT-cells enhanced NK cytotoxicity at lower extent than HC iNKTs (64). Additionally, decreased number of NKT-like cells and iNKT-cells have been reported in SLE, (65, 66) and the percentage of NKT-like cells negatively correlated with disease activity (67). In summary, our results in conjunction with other studies, provide evidence for the defective stimulation of SLE NKTs. This leads to impaired expansion, functionality and glycosylation (particularly core fucosylation and sialylation) of this subset, which in some cases, negatively correlates with disease activity indicating that these cells might play a protective role in SLE (68).

### 4.4 B-cells

In our experiments, resting plasmablasts were significantly more abundant in SLE and displayed more core fucosylated glycans than HC plasmablasts indicated by higher AAL binding, which positively correlated with SLEDAI-2K indexes. The expansion of specific plasmablast subsets (CD27^high^ as common marker) in SLE was noted in other publications as well (69, 70), however, the core fucosylation of SLE plasmablasts was proven to be unaltered (71). Overall, we observed lower degree of glycosylation on resting plasmablasts compared to other B-cell subsets. This is in line with prior findings demonstrating more truncated O-glycans on plasmablasts in comparison to other subsets (72).

In the current work, the percentage of memory B-cells within total B-cells was also significantly elevated while naive B-cells were declined and bound significantly higher Gal-3 in SLE in quiescent state. Stimulated SLE memory B-cells exhibited significantly lower binding of all the five lectins. This phenomenon was also detected, albeit not statistically significant, in naive B-cells and plasmablasts of SLE. This observation was reinforced by the higher abundance of metaclusters with low lectin binding, namely, MC02-03 in naive and MC03-05 in memory B-cells in SLE. These results are consistent with previous findings reporting reduced core fucosylation of SLE naive and memory B-cells, however sialylation was unaltered in SLE B-cell subsets (71). No further articles are currently available regarding the glycosylation and galectin binding capacity of B-cells in SLE. Therefore, a summary of relevant literature in the context of B-cells and these lectins is provided here. First, core fucosylation is identified as crucial for the antigen binding and signaling of BCR (15). Second, α-2,6-sialylation is recognized as essential for the cis-binding and oligomerization of the inhibitory Siglec, CD22, which is recruited to BCR upon activation and attenuates hyperactivation (73). Third, multiple lines of evidence highlighted the significance of Gal-3 in B-cell differentiation. Gal-3 acted as inhibitor of plasma cell differentiation (74, 75), and promoted IL-4-induced memory B-cell differentiation in Gal-3 knockout studies (76). Collectively, the literature suggests that the reduced glycosylation of SLE memory B-cells could potentially contribute to the dysregulation of BCR signaling. While the role of endogenous Gal-3 is emphasized in memory B-cell differentiation, however, the potential connection between the alterations in Gal-3 binding and the expansion of memory B-cells and plasmablasts in SLE in our results must be clarified in future research.

### 4.5 Monocytes

In the current work, monocytes were classified into phagocytic classical monocytes, pro-inflammatory non-classical monocytes, intermediate monocytes, being both phagocytic and pro-inflammatory, and transitional monocytes as the fourth and less studied MO type (77, 78). Multiple significant differences in lectin binding were observed in unstimulated monocyte subsets of HC and SLE. First, α-2,6-sialylation was decreased in classical, intermediate and non-classical monocytes, although the difference was not significant after stimulation. Cell surface sialylation was shown to be downregulated during macrophage differentiation from PMA-stimulated monocytes, as well as the expression of ST6GAL1 responsible for α-2,6-sialylation (79, 80). Liu et al. proposed that α-2,6-sialylation is protective against TNF-α-induced apoptosis in monocytes, since the downregulation of ST6GAL1 affected TNFR1 sialylation and consequently increased the level of TNF-α-induced apoptosis of PMA-stimulated monocytes (81). Speculatively, the decreased sialylation of monocyte subsets in SLE might make them more prone to apoptosis following differentiation toward macrophages.

Second, the binding of both Gal-1 and Gal-3, both have been reported to be expressed by monocytes and macrophages, were significantly reduced in resting classical MOs. According to Di Gregoli and coworkers, soluble Gal-3-negative macrophages are pro-inflammatory and Gal-3-binding macrophages promote the resolution of inflammation (82). The importance of Gal-3 binding via CD98 was underscored in alternative activation of M2 macrophages (83). Similarly, Gal-1 was also implicated in M2 macrophage polarization (84). The involvement of both pro-inflammatory M1 and nonfunctional M2 macrophages has been established in the pathogenesis of SLE (85), moreover, lupus flares were associated with M1/M2 imbalance (86). Thus, we could assume that the reduced Gal-3 and Gal-1 binding to classical MOs in SLE might dampen their ability to differentiate into pro-resolving M2 macrophages. Third, stimulated classical and intermediate MOs showed significantly lower core fucosylation in SLE.

Finally, there were various differences in the expression level of HLA-DR, CD14 and CD16 in MO subsets and their frequencies between HC and SLE. HLA-DR expression was significantly lower in resting and stimulated classical and intermediate MOs and stimulated transitional MOs in SLE. This is aligned with previous reports (87). CD14 was decreased in unstimulated classical MOs, which was also reported in SLE (87). The proportion of intermediate MOs was increased in SLE in line with other reports, where intermediate monocytes were increased in SLE compared to HC (88) or in active versus inactive SLE correlating with disease activity (89).

## 5 Conclusion

In summary, altered core fucosylation emerged as a prominent feature of SLE immune subsets. Elevated core fucosylation was evidenced in activated T-cell subsets and resting plasmablasts in SLE, correlating positively with disease activity in both cell types. Conversely, significantly enriched metaclusters in SLE within stimulated CD56^dim^ and CD56^high^ NK subsets, as identified by FlowSOM, exhibited low core fucosylation, and the frequency of these subsets positively correlated with SLEDAI-2K indices. Given the correlation of core fucosylation with disease severity in the aforementioned subsets, alterations in core fucosylation in these subsets might potentially drive pathogenic processes in SLE. On the other hand, core fucosylation was significantly decreased in stimulated DN NKTs, memory B-cells, classical MOs, and intermediate MOs in SLE. This parameter showed negative correlation with anti-ds-DNA levels in DN NKTs, suggesting a possible fucosylation-dependent protective role of these cells. Additionally, alterations in α-2,6-sialylation of immune cells were another distinctive feature of SLE. The activation-dependent increase in SNA binding in various stimulated SLE T-cell subsets displayed positive correlation with SLEDAI-2K scores. Therefore, alfa-2,6-sialylation in these subsets might contribute to their pathogenic role in SLE. Parallel to alpha-2,6-sialylation, alpha-2,3-sialylation was also significantly increased in SLE in all stimulated T-cell subsets, and also in stimulated CD4 NKTs, CD8 NKTs. Moreover, the overrepresented metaclusters with high SNA binding bound high levels of Siglec-1 as well. However, the significantly elevated Siglec-1 binding in SLE in these immune subsets did not show any correlation with disease activity. Taken these results, one could argue that the changes in alpha-2,6-sialylation of T-cells are more relevant than those in alpha-2,3-sialylation in the pathogenesis of SLE. In contrast, SNA binding significantly declined in resting and activated memory B-cells, stimulated DN NKTs, and resting classical, intermediate, and non-classical monocytes. The decrease in highly sialylated DN NKT metacluster MC04 in SLE, with its frequency negatively associated with anti-ds-DNA levels, further supports the hypothesis that DN NKT subsets might play a protective role in SLE. Similarly to SNA, Siglec-1 binding was significantly lower in activated memory B-cells in SLE. Furthermore, Siglec-1 CD25^+^ per CD25^-^ binding ratio was significantly decreased in stimulated DN NKTs in SLE and showed negative correlation with SLEDAI-2K indices. Finally, numerous immune subsets displayed significant variations in the binding capacity of Gal-1 and Gal-3 between HC and SLE. However, these characteristics did not obtain any correlation with disease activity.

The authors considered stimulating whole PBMC samples separately for the T-cell/NK cell or the B-cell/monocyte panels. Although, we found multiple examples for activation of whole PBMC samples before performing mass cytometry (90–93). Even in the scope of our research, autoimmunity field, this method was applied. One article carried out immunophenotyping of bulk-activated PBMC from active SLE, remission SLE and HC subjects (94). Moreover, it has been shown that stimulating isolated cell types will not model the cytokine secretion profile of a multicellular environment such as found in PBMC, since many cytokines are absent in single-cell cultures compared to the complexity observed in PBMC cultures (95). For example, IFN-α2, a cytokine involved in SLE pathogenesis (96) was only present in PBMC cultures but not in single-cell cultures.

Co-cultures of isolated cells are a closer predictor of cytokines found in PBMC cultures. Co-cultures of immune cells is a useful tool for studying interactions between the cell, although in such experiments, the ratio of T-cells and APCs is relatively high such as 3: 1, 1: 1 or lower, 1: 2 -1: 5. In comparison, the estimated starting ratio of cells in our PBMC cultures based on the data of the two panels is 1:13.8 for B-cell/T-cell and 1: 8.6 for monocyte/T-cell, with no significant difference between HC and SLE. So arguably, the interaction between cells is inevitable in PBMC cultures but it is less likely that the significant differences discovered in our study are a consequence of uncontrolled interactions between different cell types due to naturally varying ratios of cell types within PBMC samples. Indeed, in our data no correlation between CD25^+^ positivity within T-cells and the ratios of B-cell/T-cell and monocyte/T-cell has been identified (**Supplementary Figure 3**). Furthermore, when B-cells were stimulated by ODN2006 in whole PBMC culture, but T-cells were not, the frequency of CD25^+^ cells within CD3^+^ T-cells did not change. Therefore, it is unlikely that unstimulated B-cells provide additional activation stimulus or inhibition to T-cells in the co-culture (**Supplementary Figure 3**). It has been demonstrated that low-dose IL-2 can promote the differentiation of regulatory B-cells, although CpG pre-stimulation (ODN2395) is required for the induction of IL-10 producing B-cells by low dose IL-2 which could inhibit the TNF-α production of CD4 T-cells in a co-culture of B-cells and T-cells in 1: 5 ratio (97). Accordingly, in our experiments CpG stimulation (ODN2006) in combination with CytoStim (CS) and IL-2 decreased CD25-positivity of T-cells by only 9% compared to CS+IL-2 stimulated T-cells (71.9 vs. 62.9%). This is a relatively small inhibition, and we have to emphasize that the literature also suggests that for this inhibitory effect, B-cells must be pre-stimulated by a combination of low-dose IL-2 and CpG. Additionally, the authors think that separate investigation of individual cell types could be far from the *in vivo* situation where cell-cell interactions occur. Therefore, in line with the above literature data, the authors rely more on the results based on PBMCs instead of separate cell types.

The descriptive nature of our findings can be regarded as a limitation of the study. However, our aim to identify potentially disease-specific immune cell subsets as characterized by their lectin-binding affinity and activation status could be achieved with this approach. We intended to exploit the advantages of multi-channel flow cytometry and the advanced bioinformatic analysis of the data obtainable from the samples. We plan to perform further characterization of the identified cell subsets and we also hope that the cell populations emerging in this research can be helpful to further research in this field.

Another limitation may be the administration of maintenance immunosuppression in many of the patients. However, the major inclusion criterion was that SLE must be active, and we think that the immunological processes of active lupus can well be characterized in this cohort as the clinical situation reflected that these processes had been activated despite immunosuppressive therapy with maintenance doses. Also, for this purpose, high intensity immunosuppression and prednisolone at a dose > 5 mg were applied as exclusion criteria.

Taken together, our study identified numerous glycan alterations in several immune subsets in SLE associated with disease activity. These changes may be representing relevant and novel factors in the pathogenesis of SLE, even though they reflect the whole cell surface of the cells and not specific proteins. To gain a more comprehensive understanding, it would be beneficial to identify the exact glycoproteins or glycolipids involved in the altered cell surface glycome of SLE immune cells.

## Data availability statement

The raw data supporting the conclusions of this article will be made available by the authors, without undue reservation.

## Ethics statement

Details about the study design and handling of biological materials were submitted to the Human Investigation Review Board of the University of Szeged under the 21/2011 and 149/2019-SZTE Project Identification codes. The studies were conducted in accordance with the local legislation and institutional requirements. The participants provided their written informed consent to participate in this study.

## Author contributions

ES: Conceptualization, Data curation, Formal analysis, Investigation, Methodology, Software, Writing – original draft, Writing – review & editing. AF: Investigation, Writing – original draft. GB: Investigation, Writing – original draft. NG: Writing – original draft, Writing – review & editing, Investigation. LP: Conceptualization, Resources, Writing – original draft. LK: Conceptualization, Data curation, Resources, Supervision, Writing – original draft, Writing – review & editing. GS: Conceptualization, Data curation, Resources, Supervision, Writing – original draft, Writing – review & editing.
