# Supplementary material for: Identification of immune subsets with distinct lectin binding signatures using multi-parameter flow cytometry: correlations with disease activity in systemic lupus erythematosus
Source: Front Immunol. 2024 May 7;15:1380481. doi: 10.3389/fimmu.2024.1380481 (PMC11106380; doi:10.3389/fimmu.2024.1380481)
Supplement: Supplementary file 1 [file DataSheet_1.pdf]

Compensation
of
T/NK/NKT-cell panel

Unstained

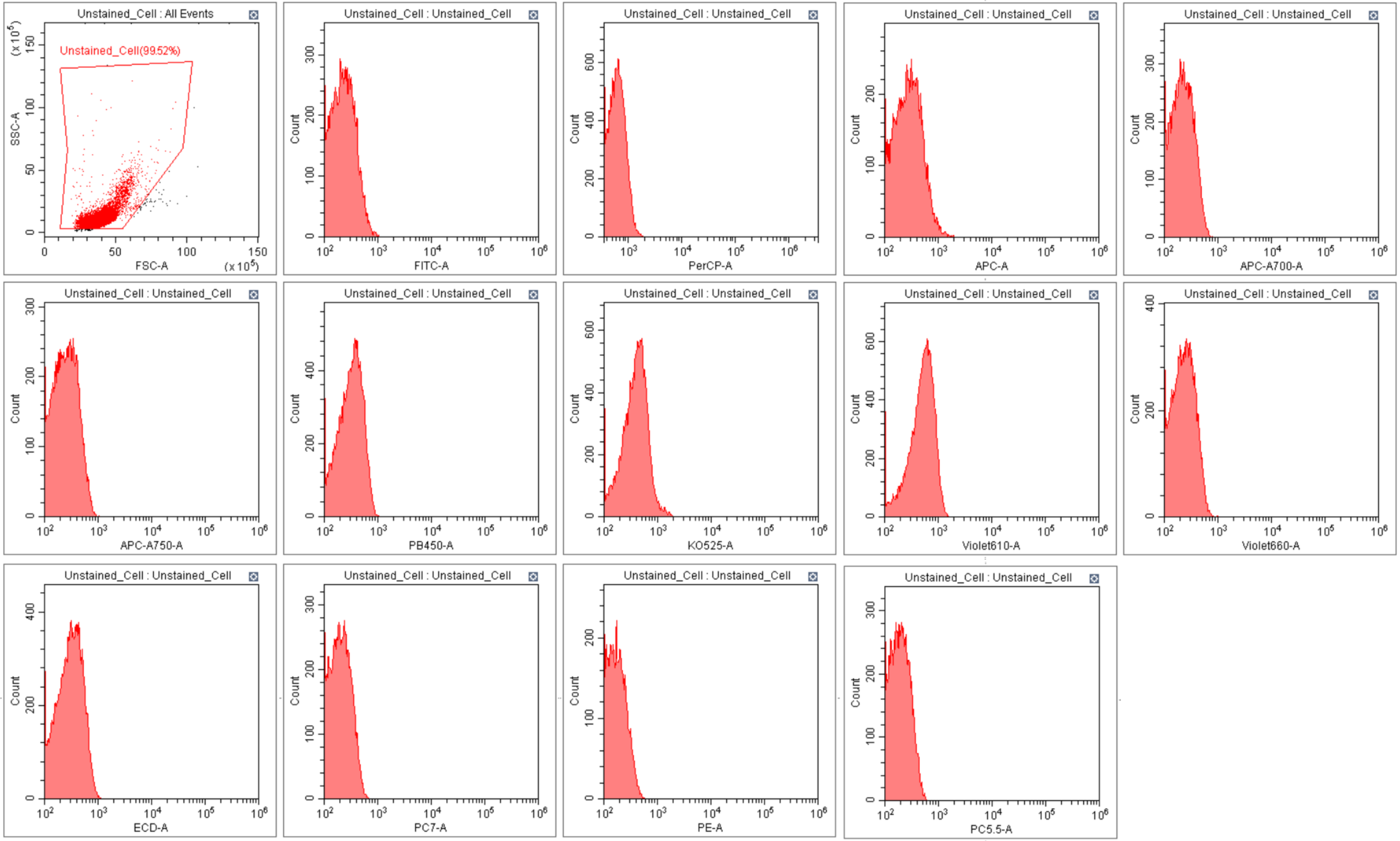

Unstained_Cell : All Events
Unstained_Cell(99.52%)
SSC-A
(x 10^5)
FSC-A
(x 10^5)
Unstained_Cell : Unstained_Cell
Count
FITC-A
PerCP-A
APC-A
APC-A700-A
APC-A750-A
PB450-A
KO525-A
Violet610-A
Violet660-A
ECD-A
PC7-A
PE-A
PC5.5-A

SNA-FITC

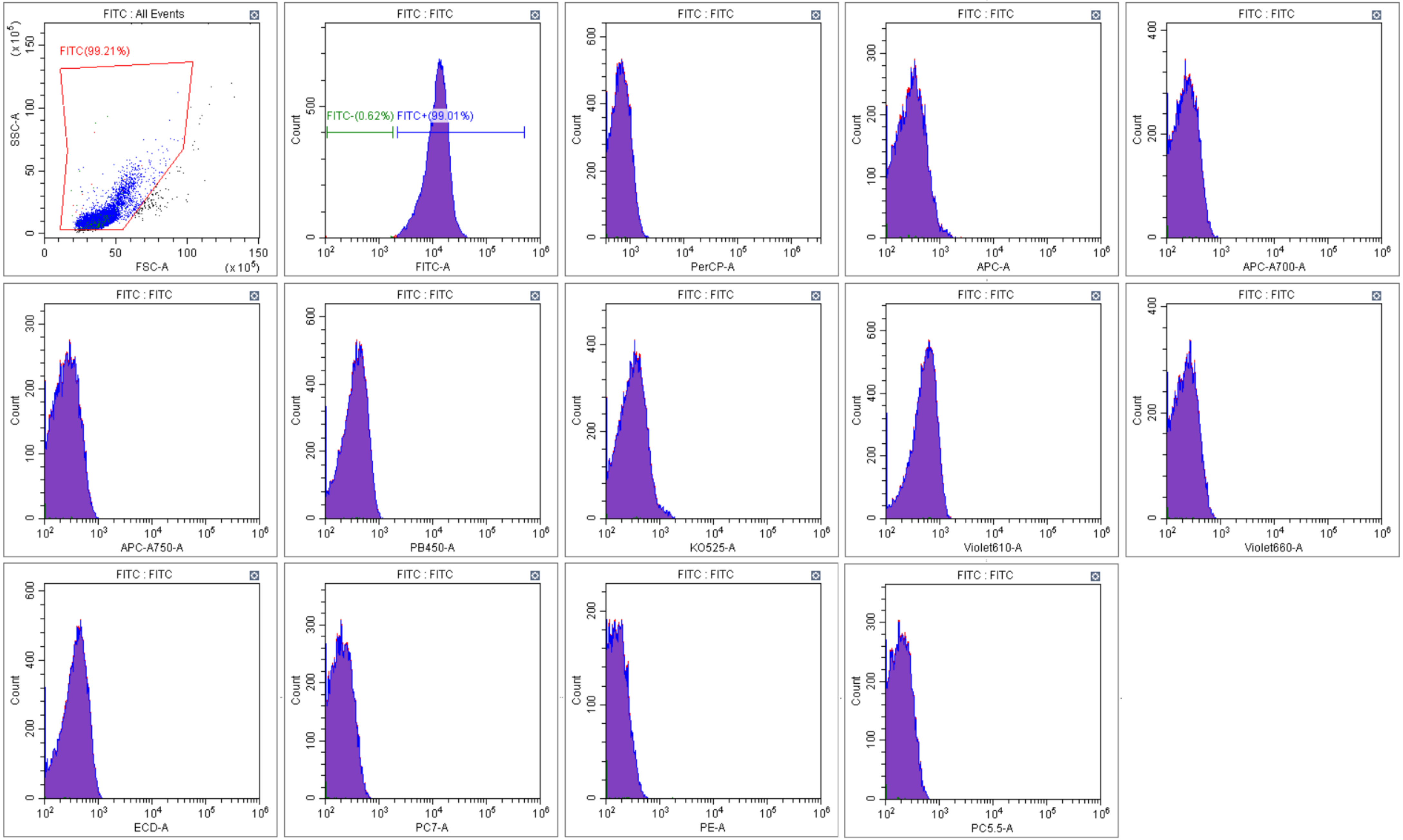
FITC : All Events
FITC(99.21%)
SSC-A
FSC-A
FITC : FITC
FITC-(0.62%)
FITC+(99.01%)
Count
FITC-A
PerCP-A
APC-A
APC-A700-A
APC-A750-A
PB450-A
KO525-A
Violet610-A
Violet660-A
ECD-A
PC7-A
PE-A
PC5.5-A

CD8-PerCP

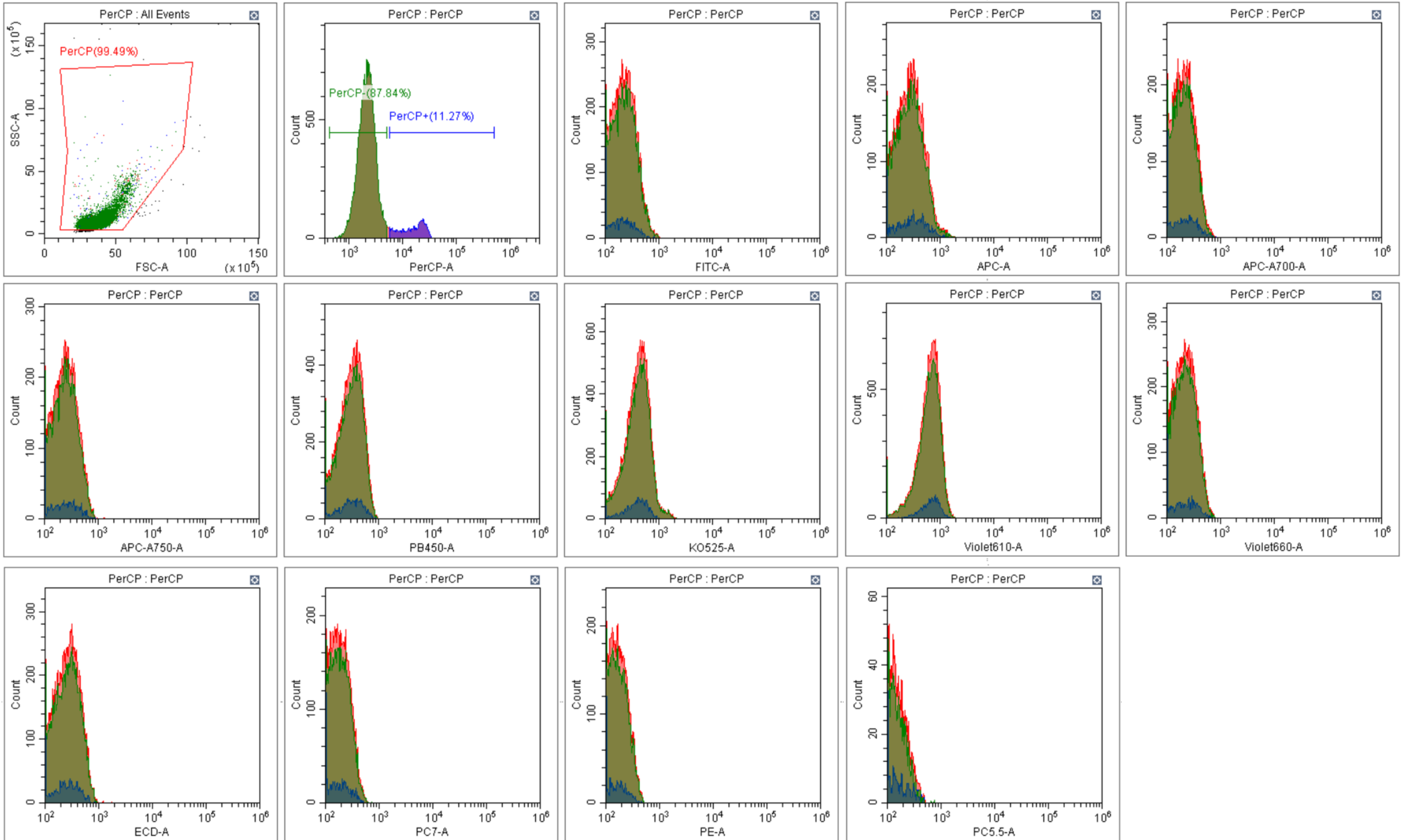

PerCP : All Events
PerCP(99.49%)
SSC-A (x 10^5)
FSC-A (x 10^5)
PerCP : PerCP
PerCP-(87.84%)
PerCP+(11.27%)
Count
PerCP-A
FITC-A
APC-A
APC-A700-A
APC-A750-A
PB450-A
KO525-A
Violet610-A
Violet660-A
ECD-A
PC7-A
PE-A
PC5.5-A

AAL-APC

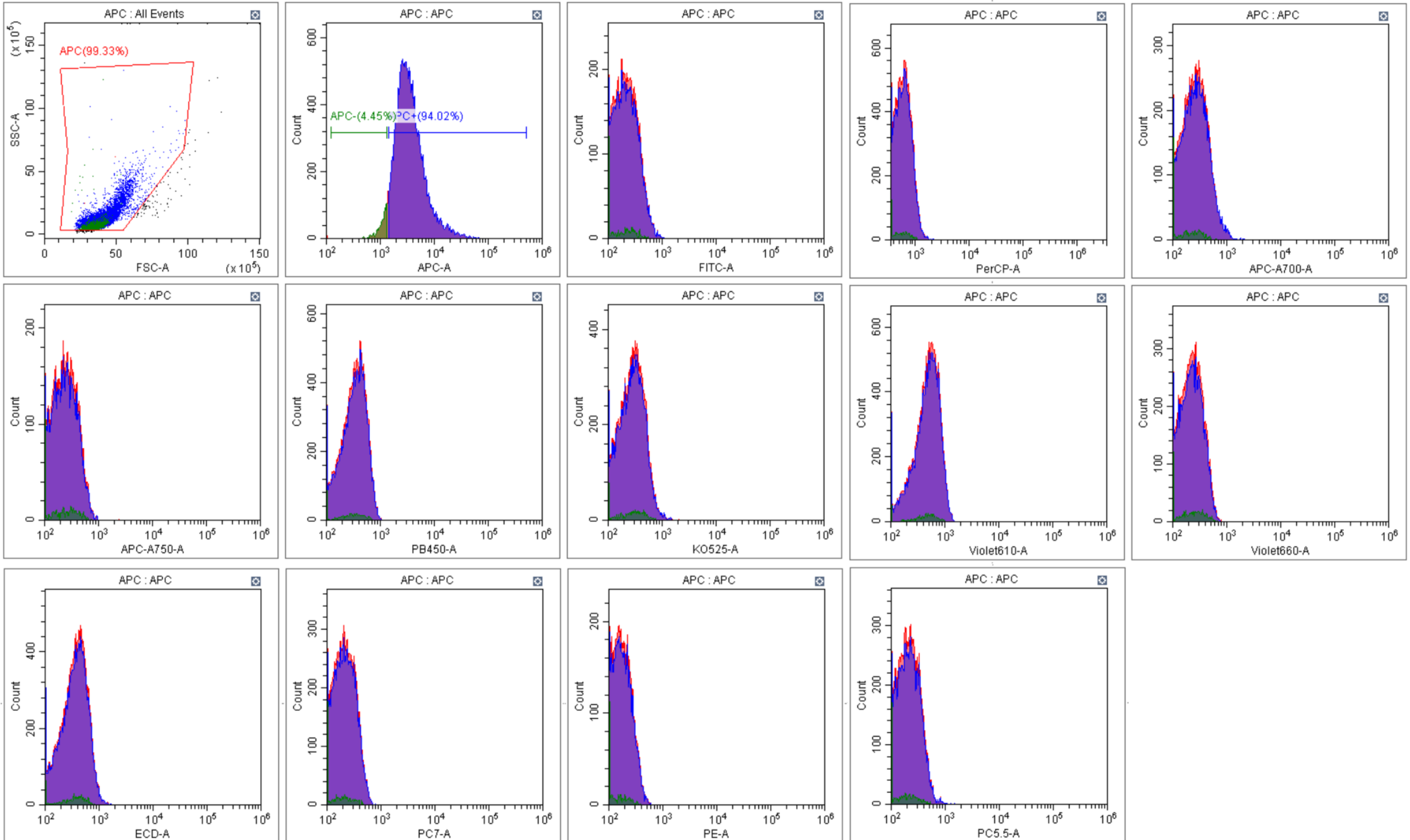
APC : All Events
APC(99.33%)
SSC-A
FSC-A
APC : APC
APC-(4.45%)
PC+(94.02%)
Count
APC-A
FITC-A
PerCP-A
APC-A700-A
APC-A750-A
PB450-A
KO525-A
Violet610-A
Violet660-A
ECD-A
PC7-A
PE-A
PC5.5-A

CD4-AlexaFluor700

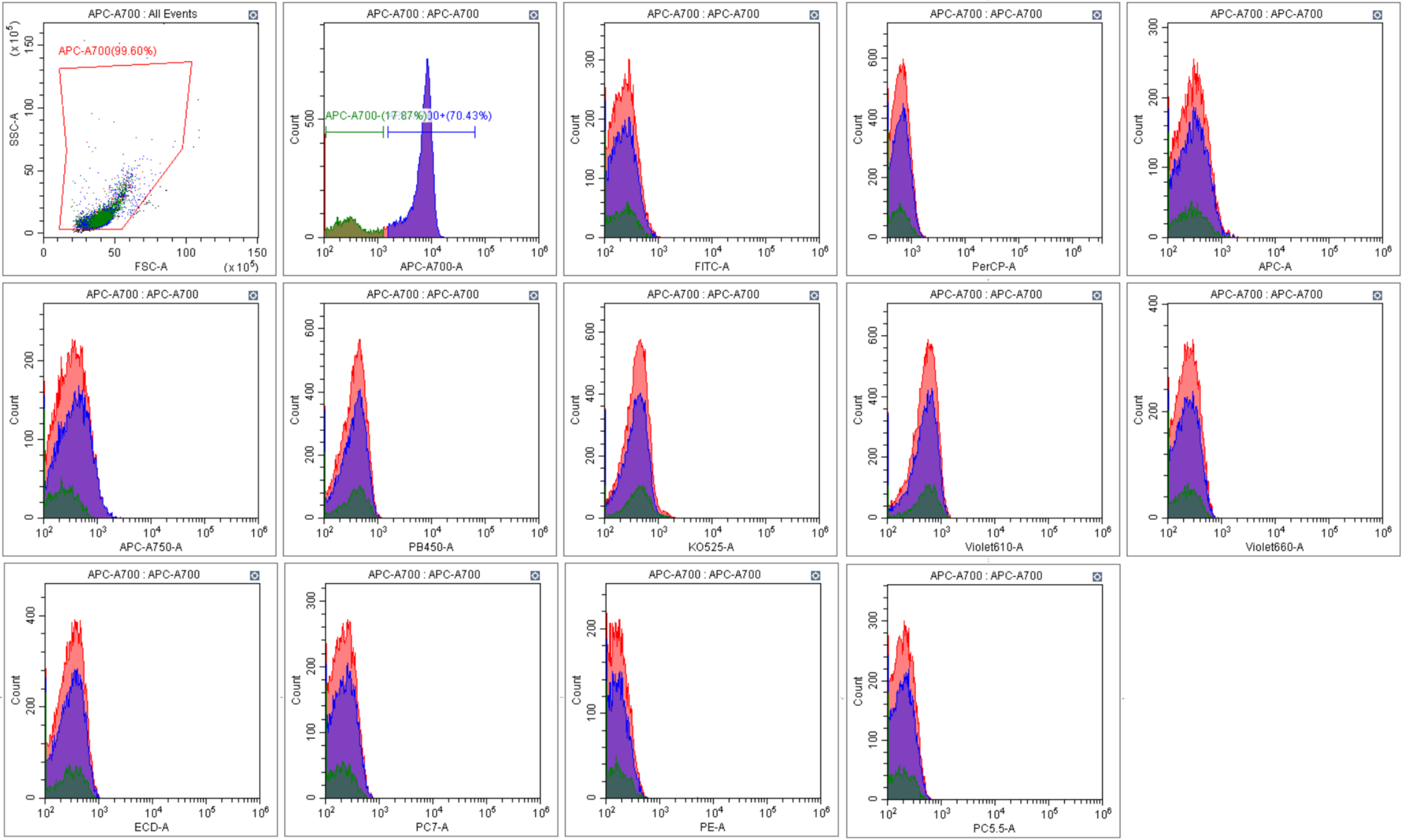

Gal-3 APC-Cy7

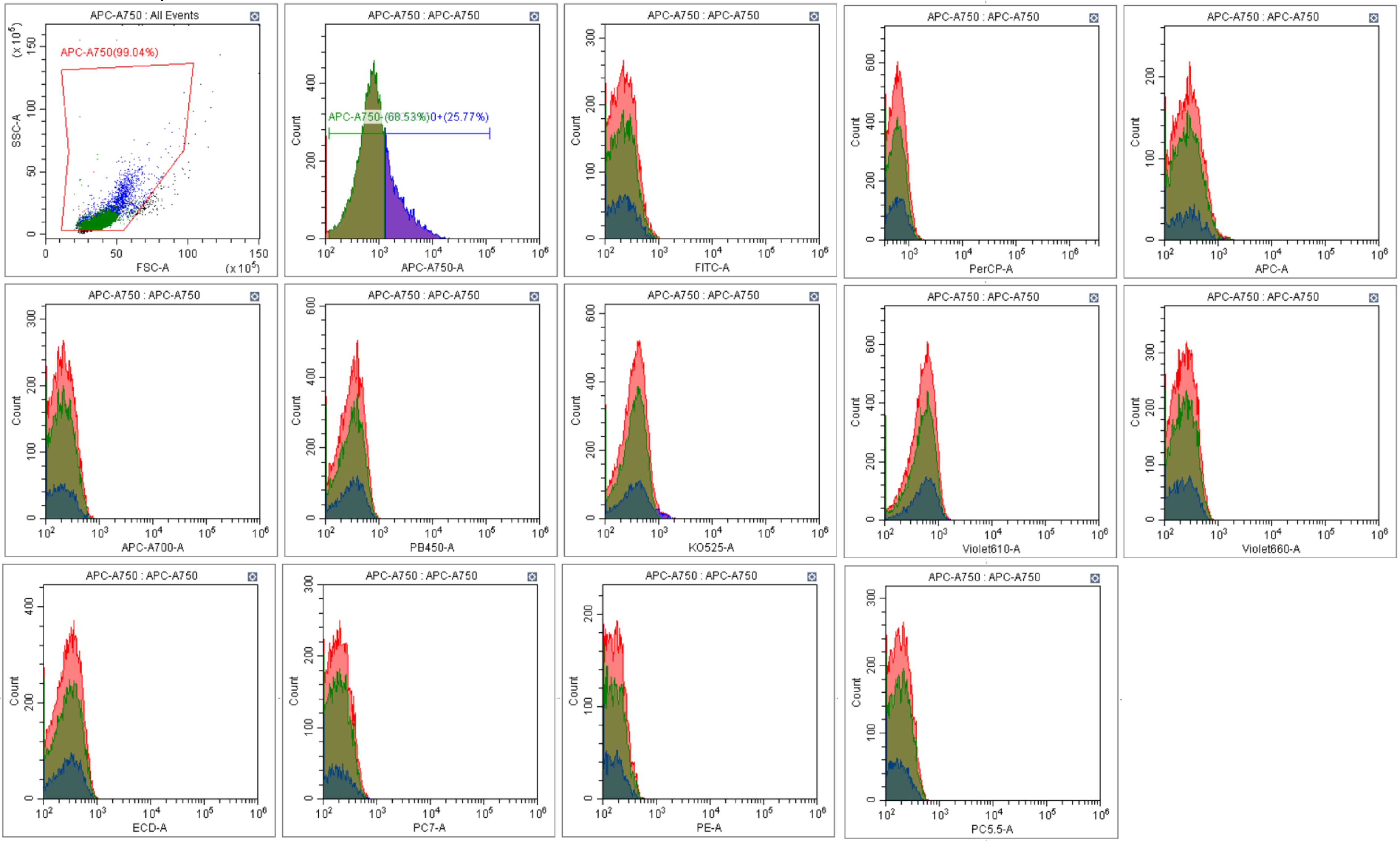
APC-A750 : All Events
APC-A750(99.04%)
SSC-A
FSC-A
(x 10^5)
APC-A750 : APC-A750
APC-A750-(68.53%)0+(25.77%)
Count
APC-A750-A
FITC-A
PerCP-A
APC-A
APC-A700-A
PB450-A
KO525-A
Violet610-A
Violet660-A
ECD-A
PC7-A
PE-A
PC5.5-A

CD3-eFluor450

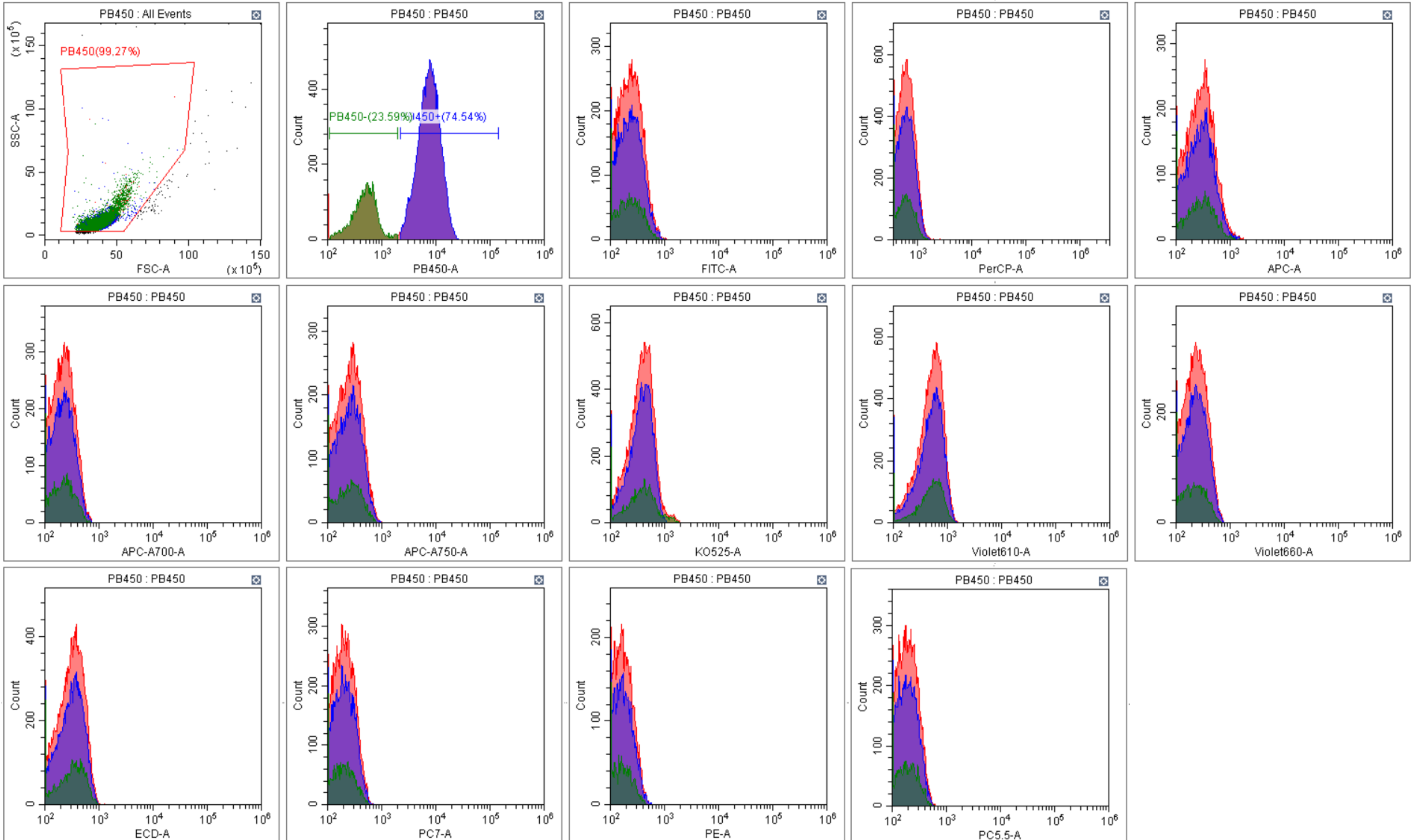

Viobility 405/520

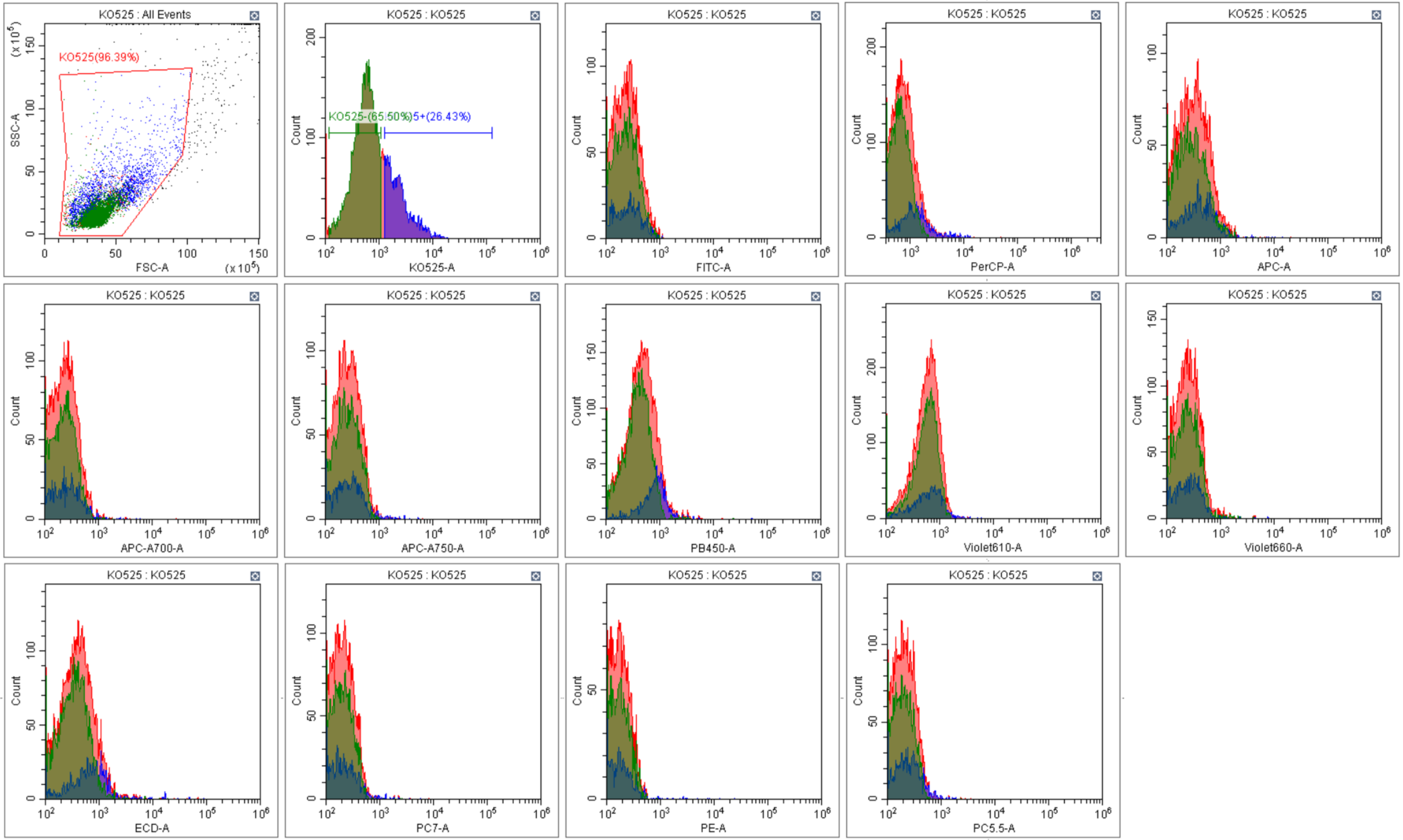

CD56-Brilliant Violet 605

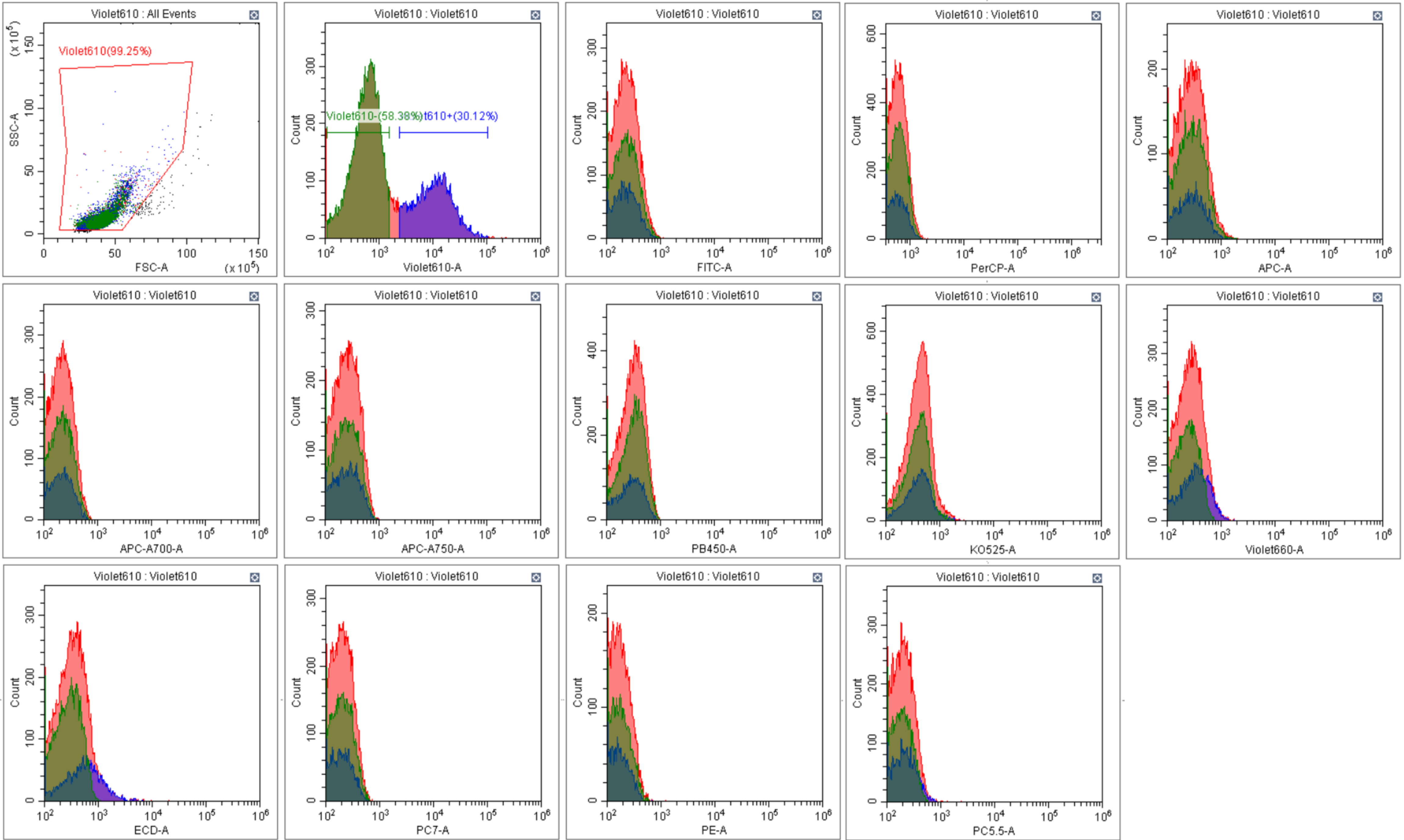

CD25-Super Bright 645

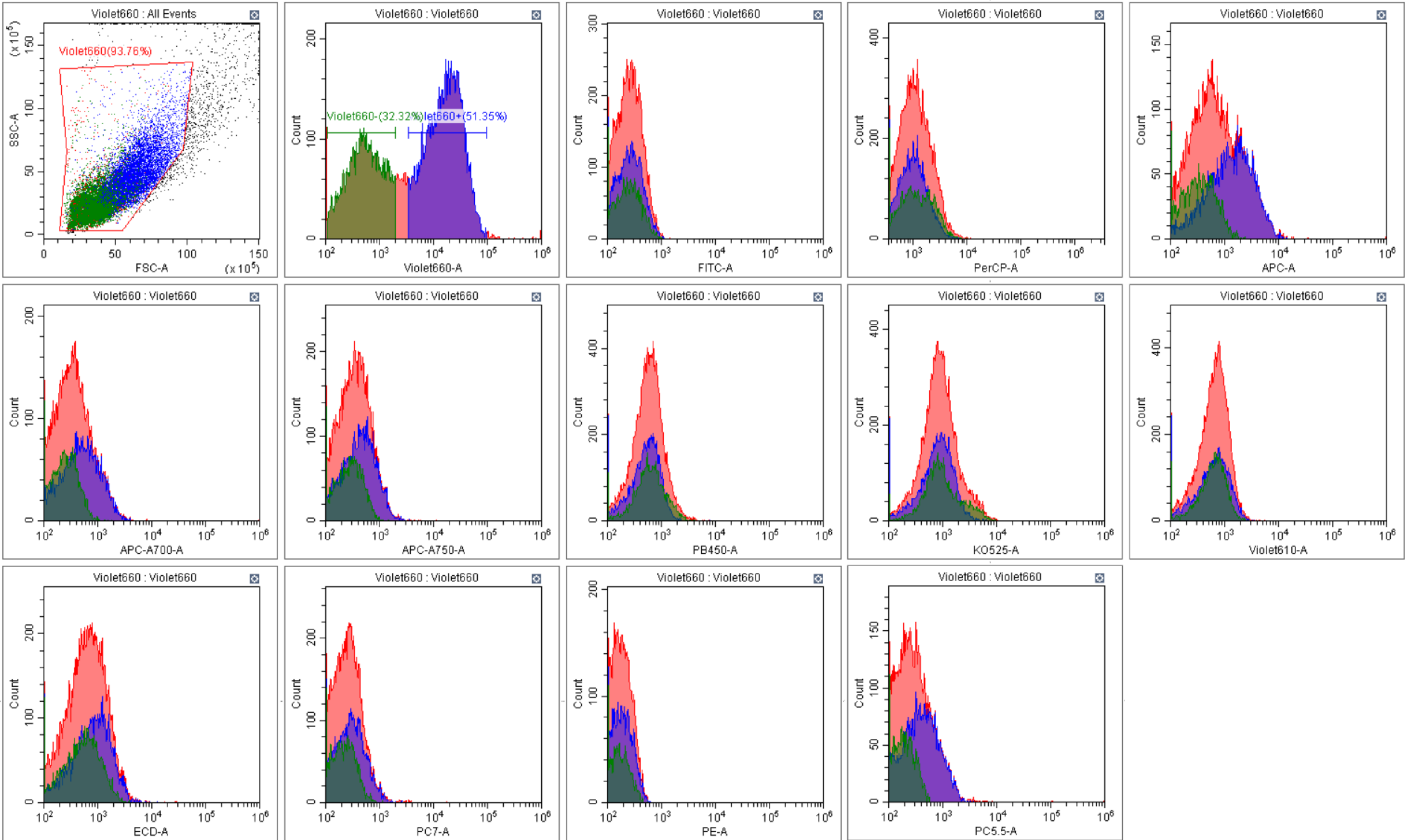

Siglec-1 PE-Texas Red

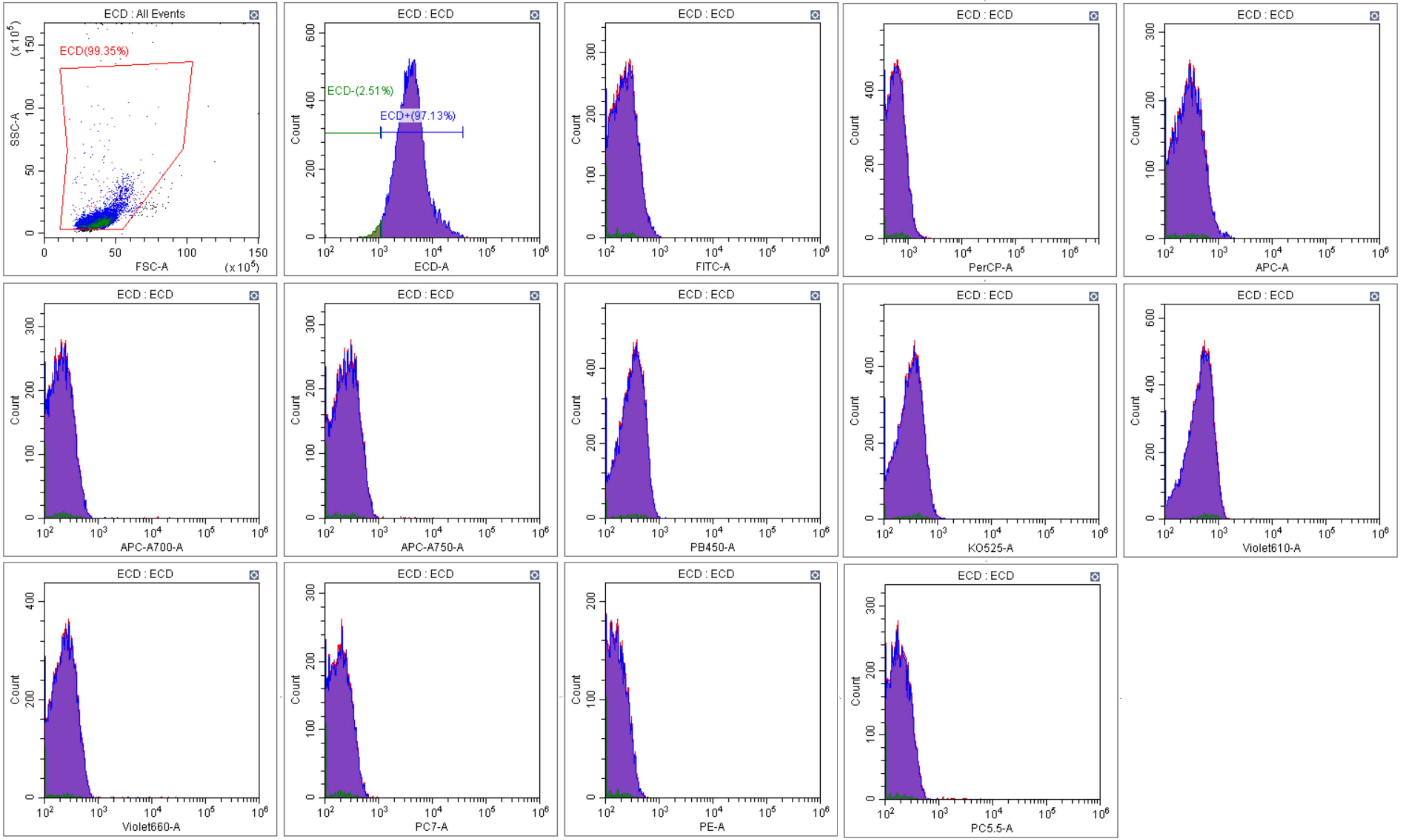

Gal-1 PE-Cy7

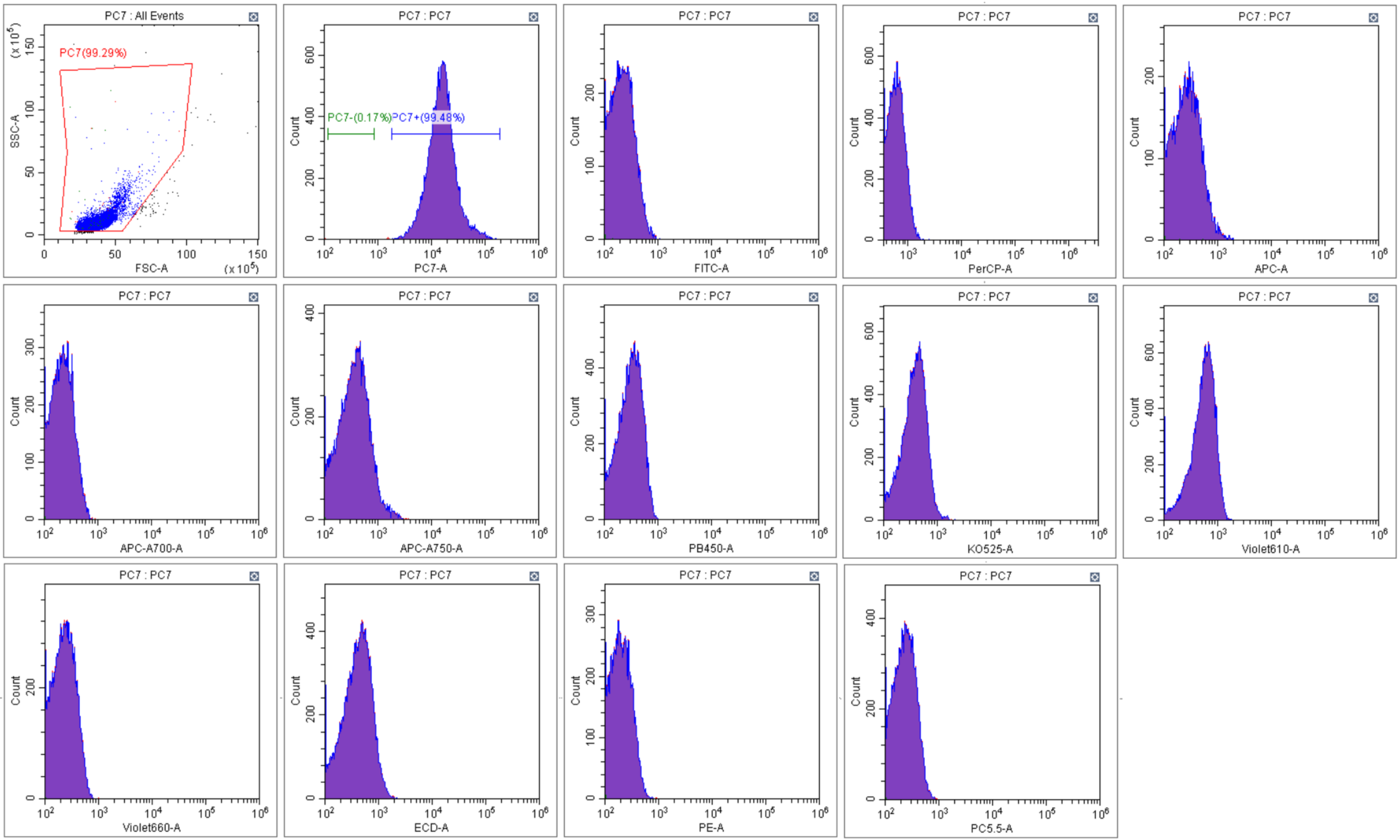

PC7 : All Events
PC7(99.29%)
SSC-A
FSC-A
PC7 : PC7
PC7-(0.17%)
PC7+(99.48%)
Count
PC7-A
FITC-A
PerCP-A
APC-A
APC-A700-A
APC-A750-A
PB450-A
KO525-A
Violet610-A
Violet660-A
ECD-A
PE-A
PC5.5-A

Compensation
of
B-cell/monocyte panel

Unstained sample

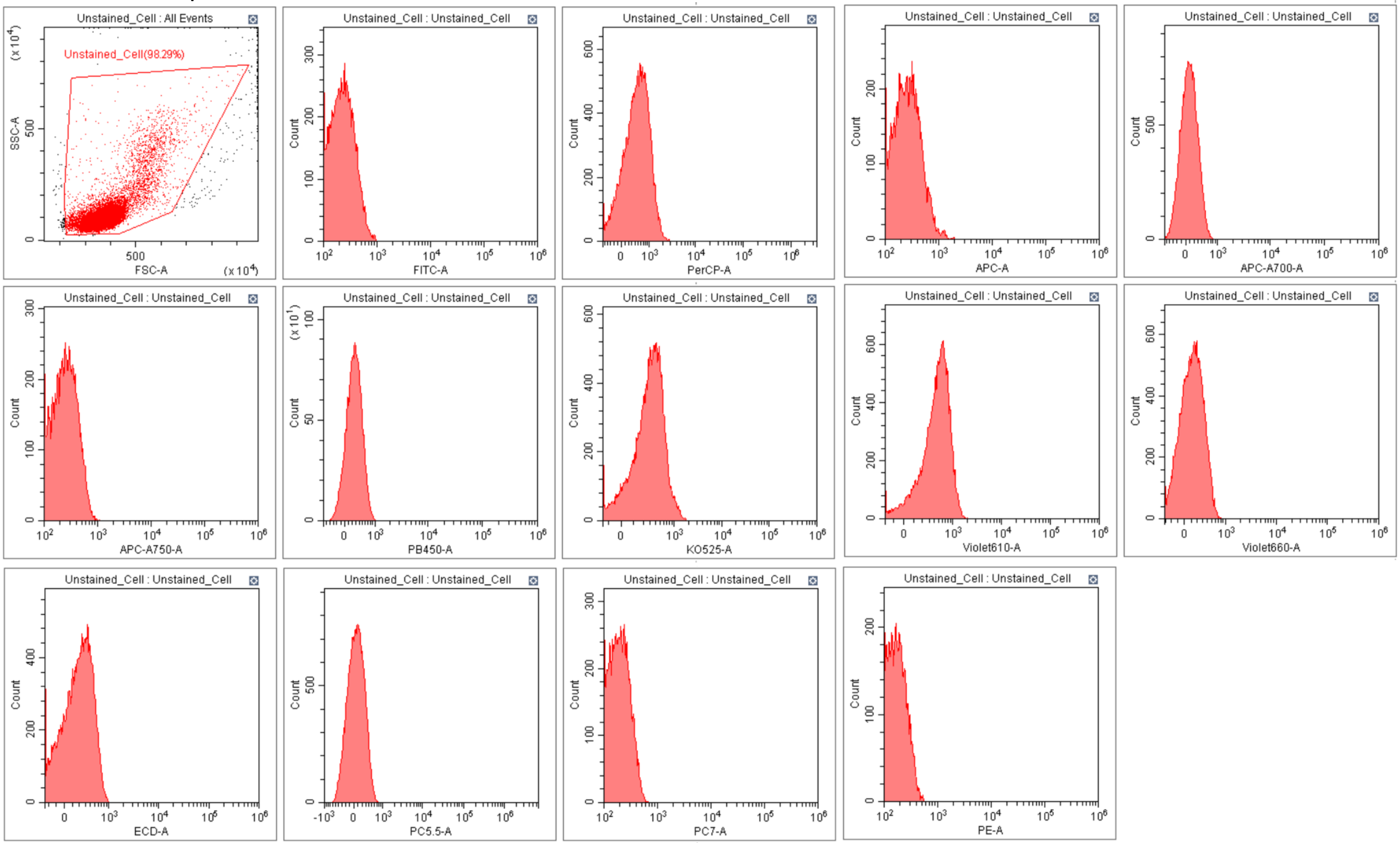

SNA-FITC

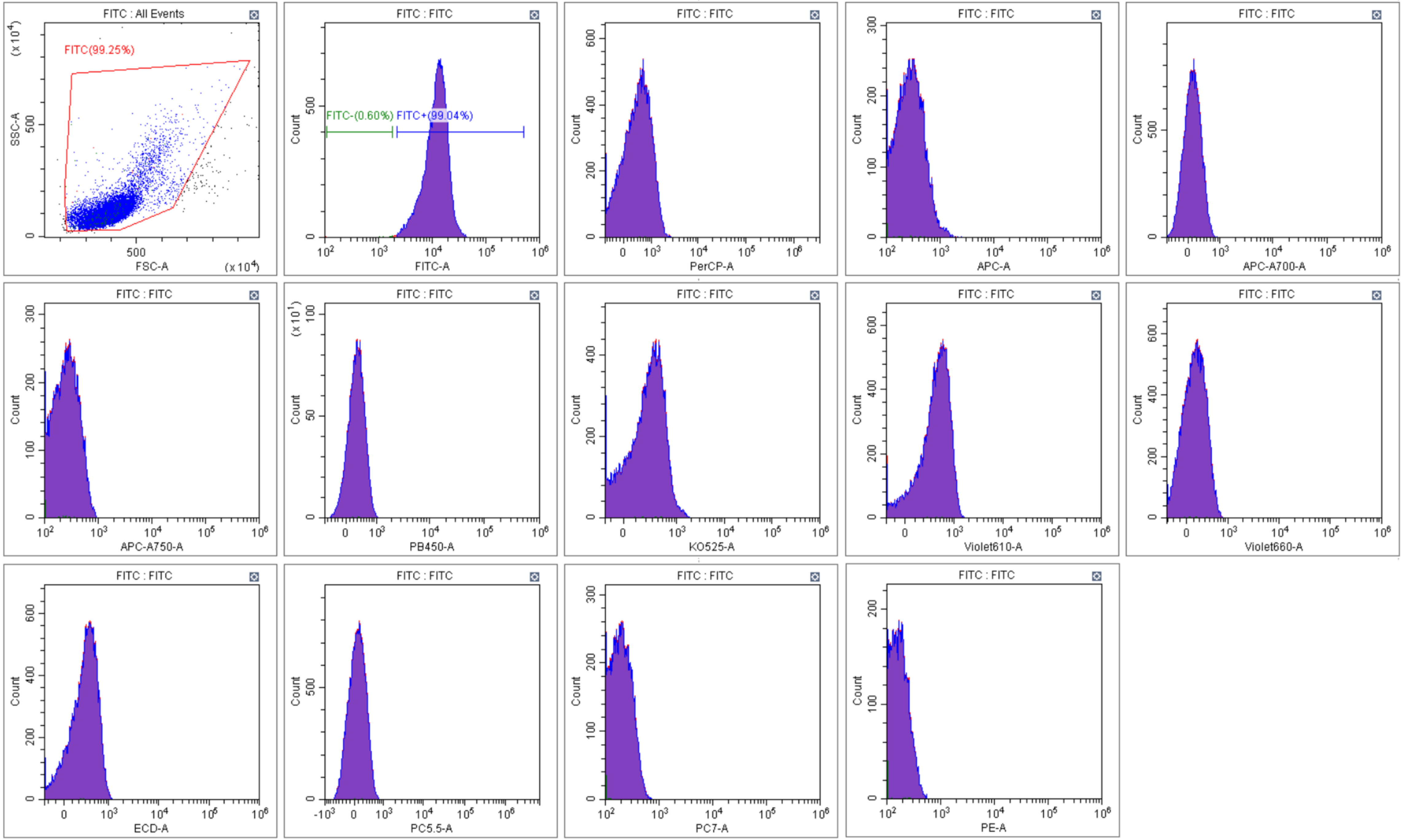
FITC : All Events
FITC(99.25%)
SSC-A
FSC-A
FITC : FITC
FITC-(0.60%) FITC+(99.04%)
Count
FITC-A
PerCP-A
APC-A
APC-A700-A
APC-A750-A
PB450-A
KO525-A
Violet610-A
Violet660-A
ECD-A
PC5.5-A
PC7-A
PE-A

CD38-PerCP

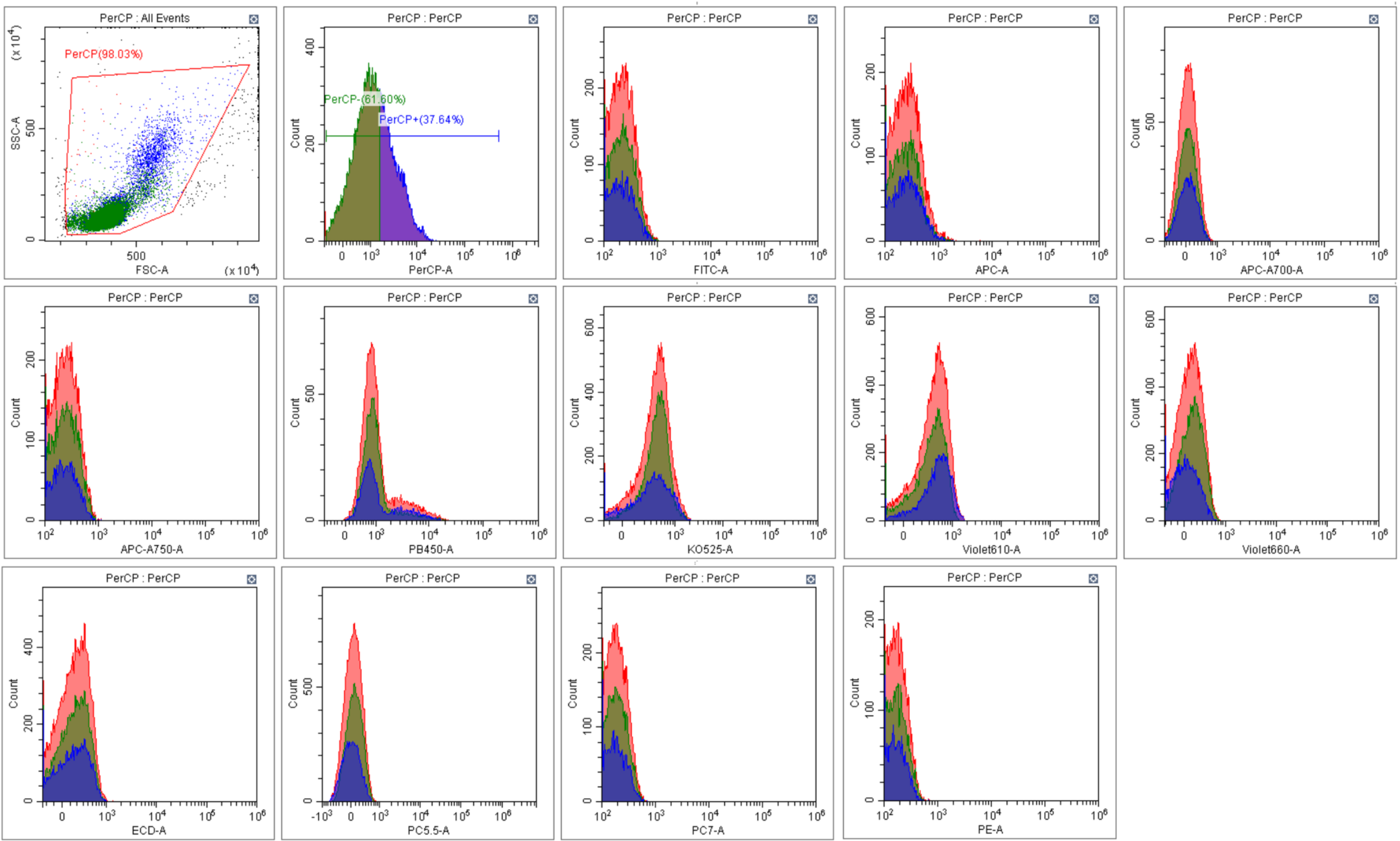

PerCP : All Events
PerCP(98.03%)
SSC-A
FSC-A
PerCP : PerCP
PerCP-(61.60%)
PerCP+(37.64%)
Count
PerCP-A
FITC-A
APC-A
APC-A700-A
APC-A750-A
PB450-A
KO525-A
Violet610-A
Violet660-A
ECD-A
PC5.5-A
PC7-A
PE-A

AAL-APC

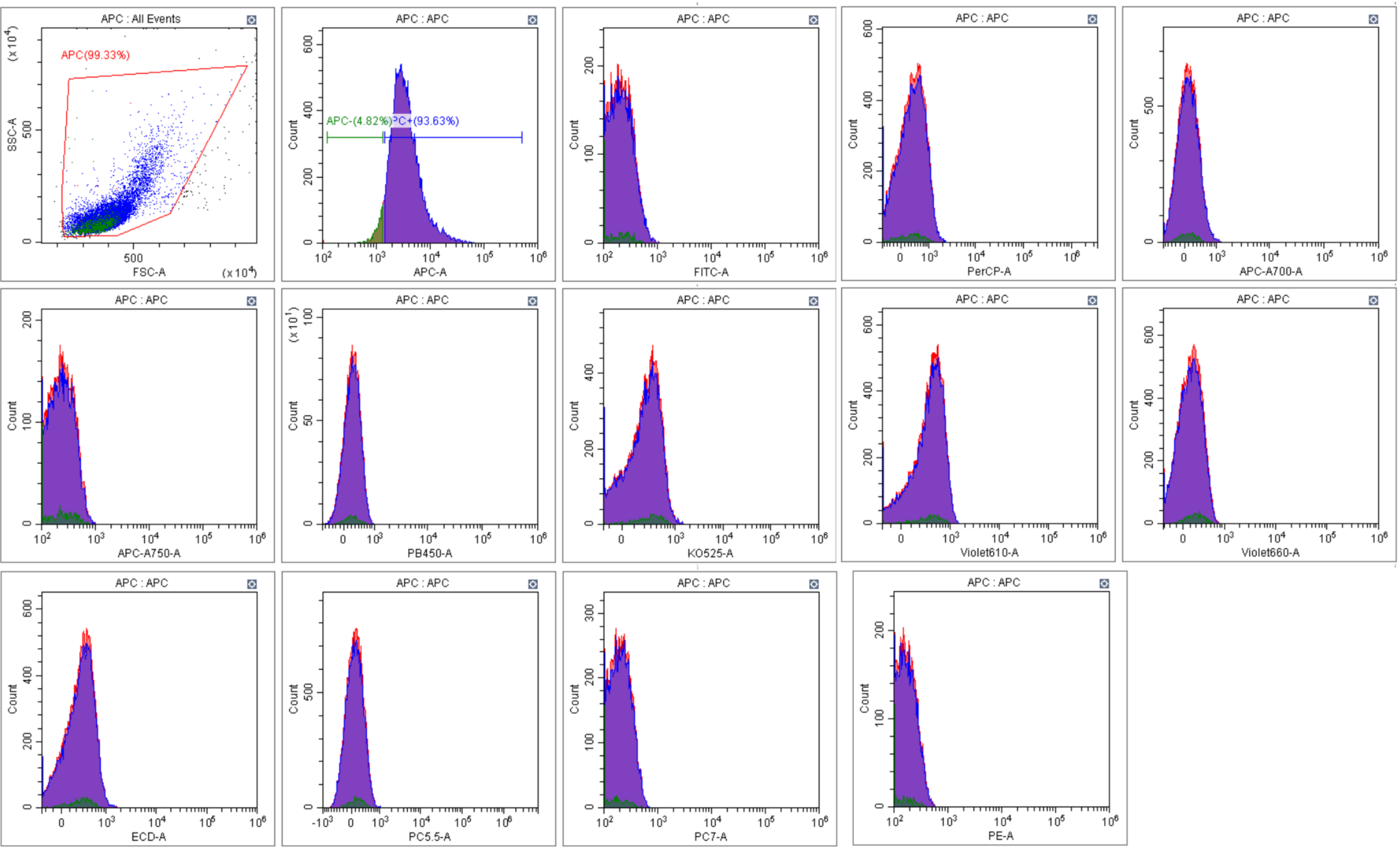

APC : All Events
APC(99.33%)
SSC-A
FSC-A
(x 10^4)
APC : APC
APC-(4.82%)
PC+(93.63%)
Count
APC-A
FITC-A
PerCP-A
APC-A700-A
APC-A750-A
PB450-A
KO525-A
Violet610-A
Violet660-A
ECD-A
PC5.5-A
PC7-A
PE-A

CD16-AlexaFluor700

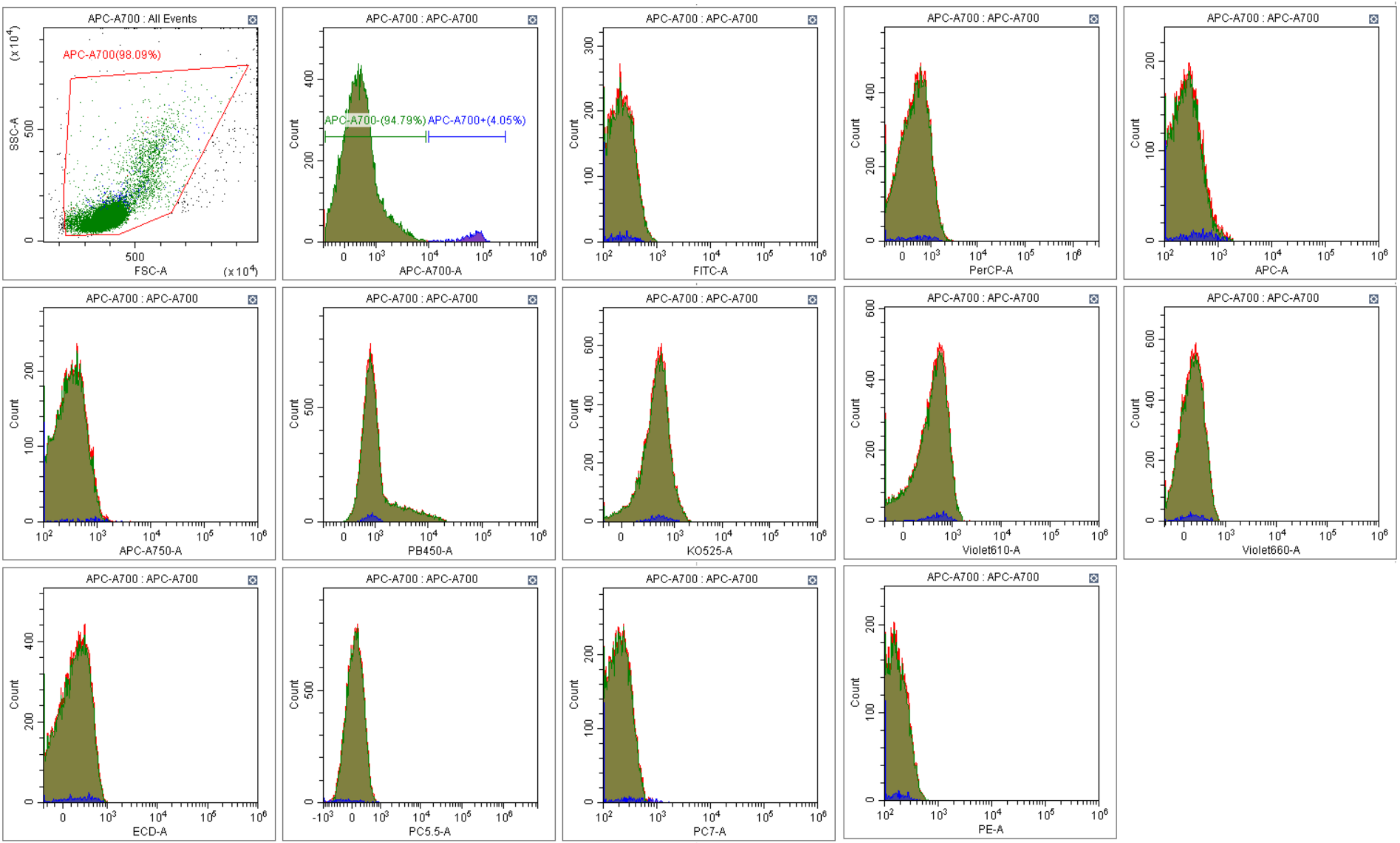

Gal-3 APC-Cy7

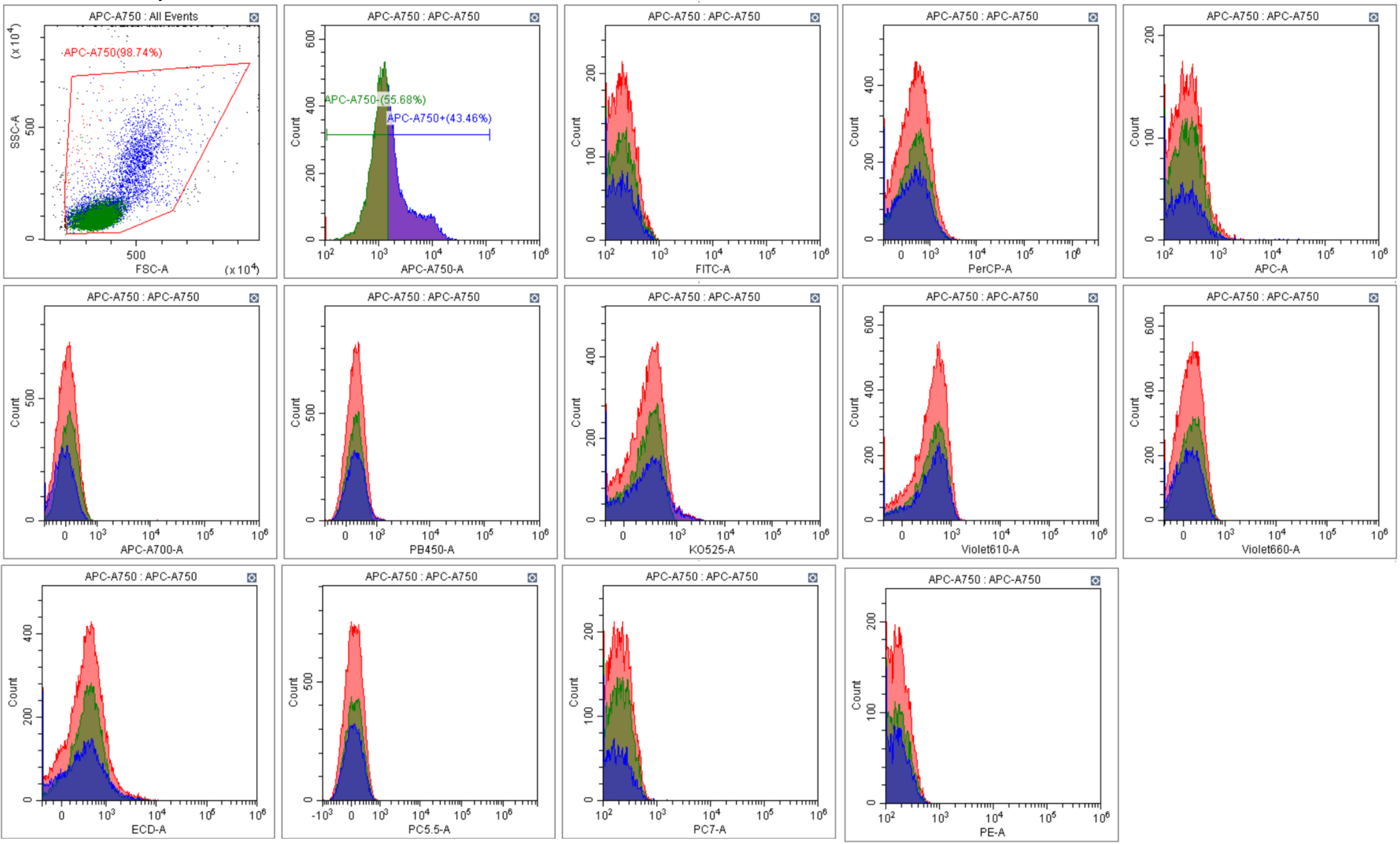

APC-A750 : All Events
APC-A750(98.74%)
SSC-A
FSC-A
APC-A750 : APC-A750
APC-A750-(55.68%)
APC-A750+(43.46%)
Count
APC-A750-A
FITC-A
PerCP-A
APC-A
APC-A700-A
PB450-A
KO525-A
Violet610-A
Violet660-A
ECD-A
PC5.5-A
PC7-A
PE-A

CD27-Pacific Blue

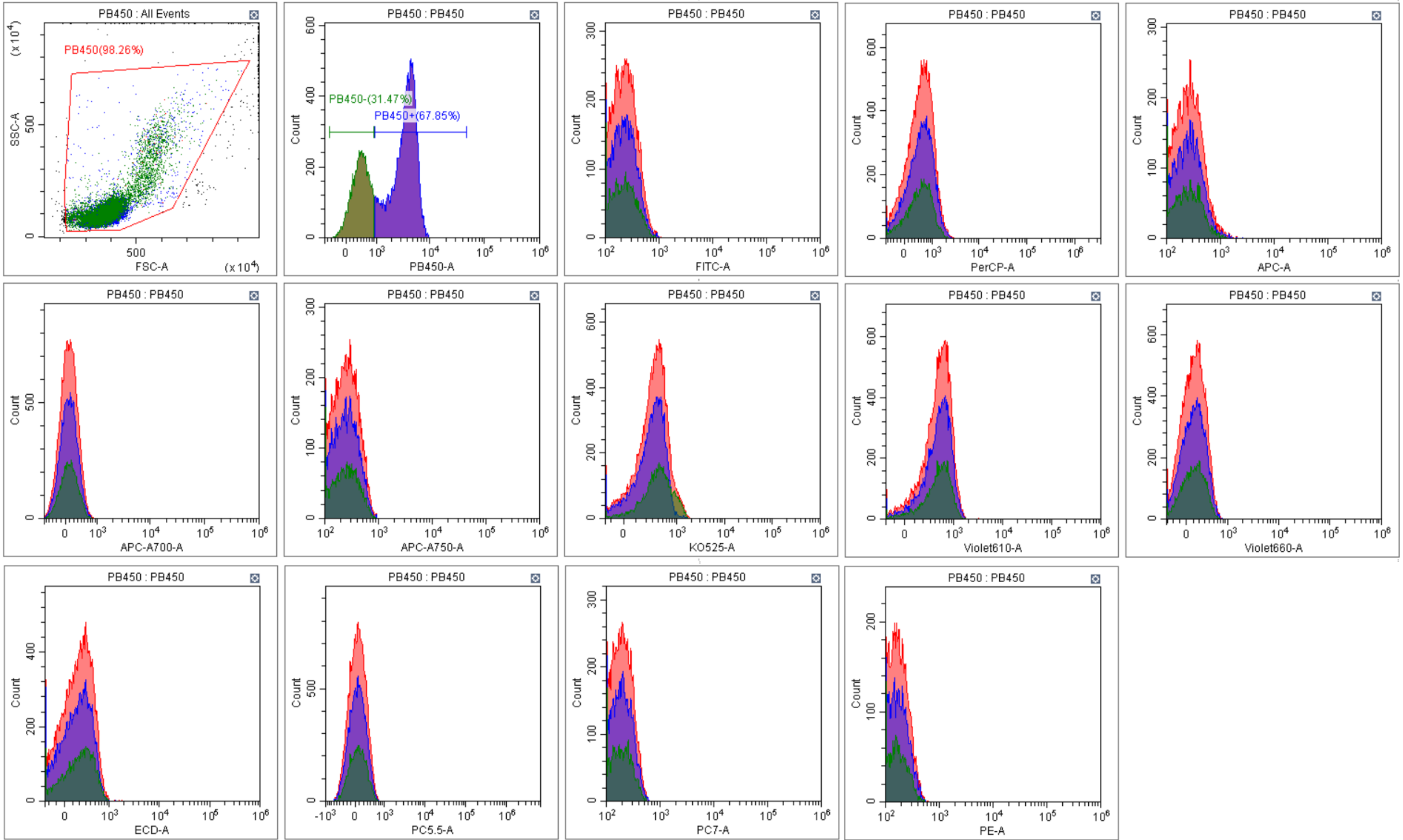

Viobility 405/520

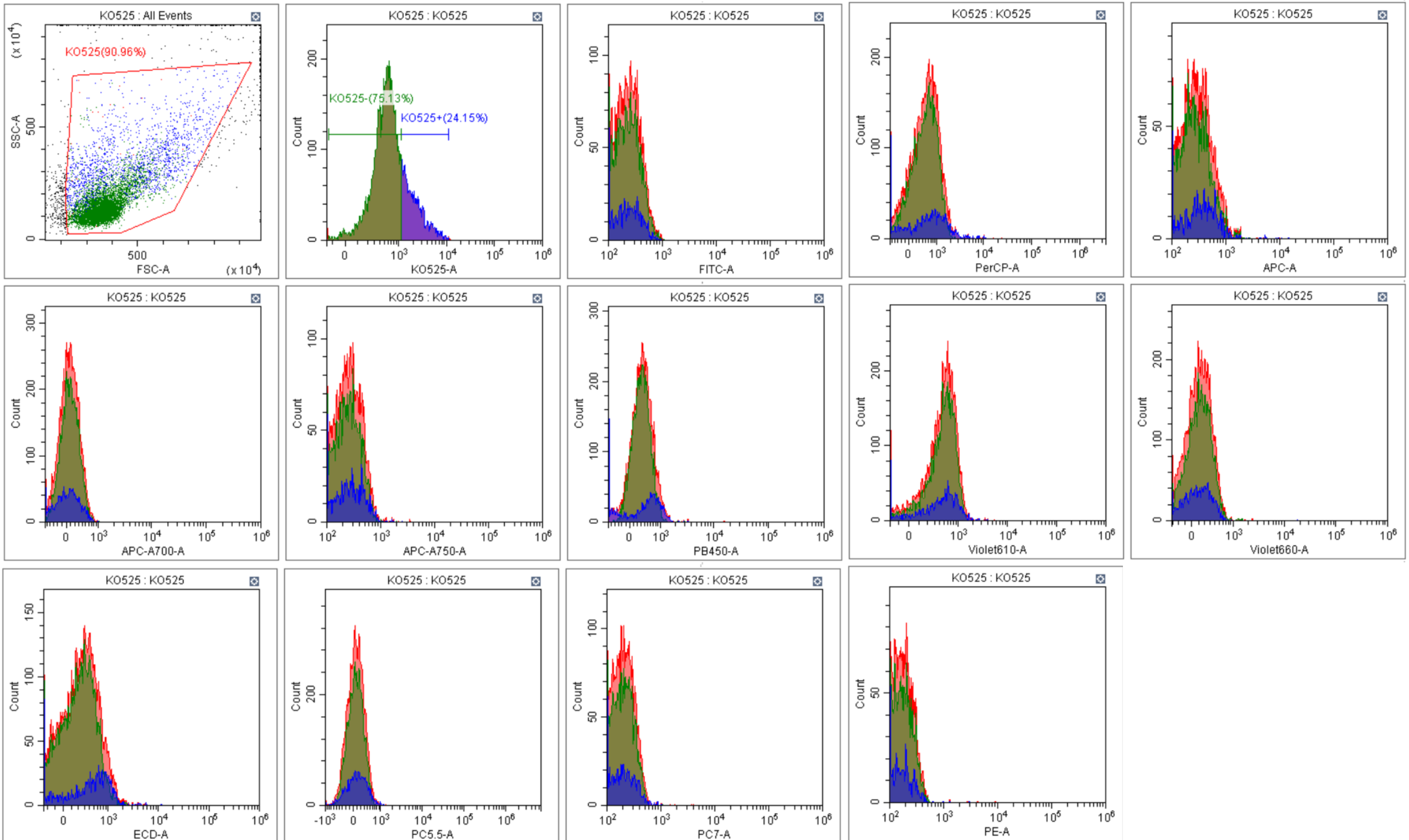

CD19-Brilliant Violet 605

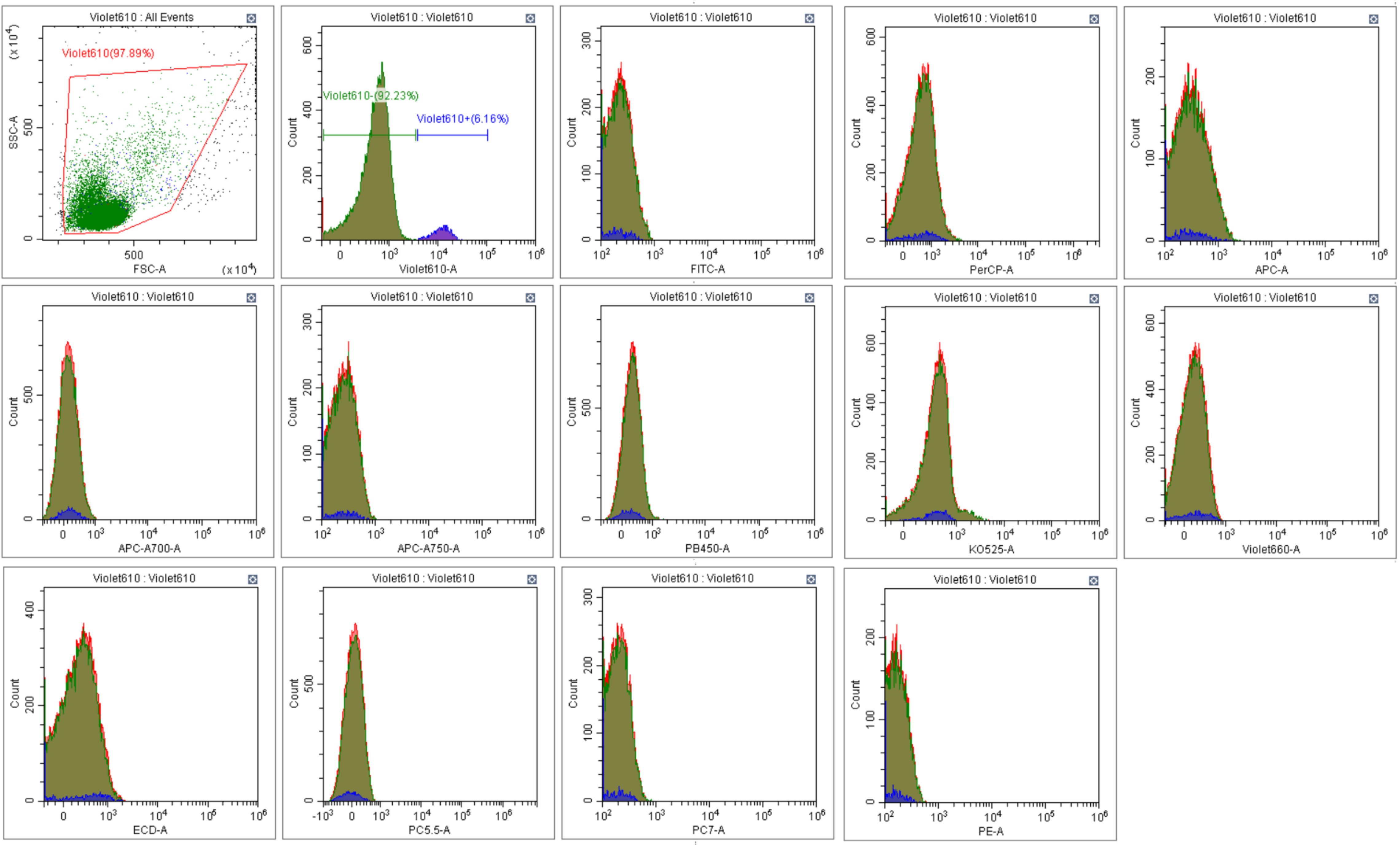

CD14-Brialliant Violet 650

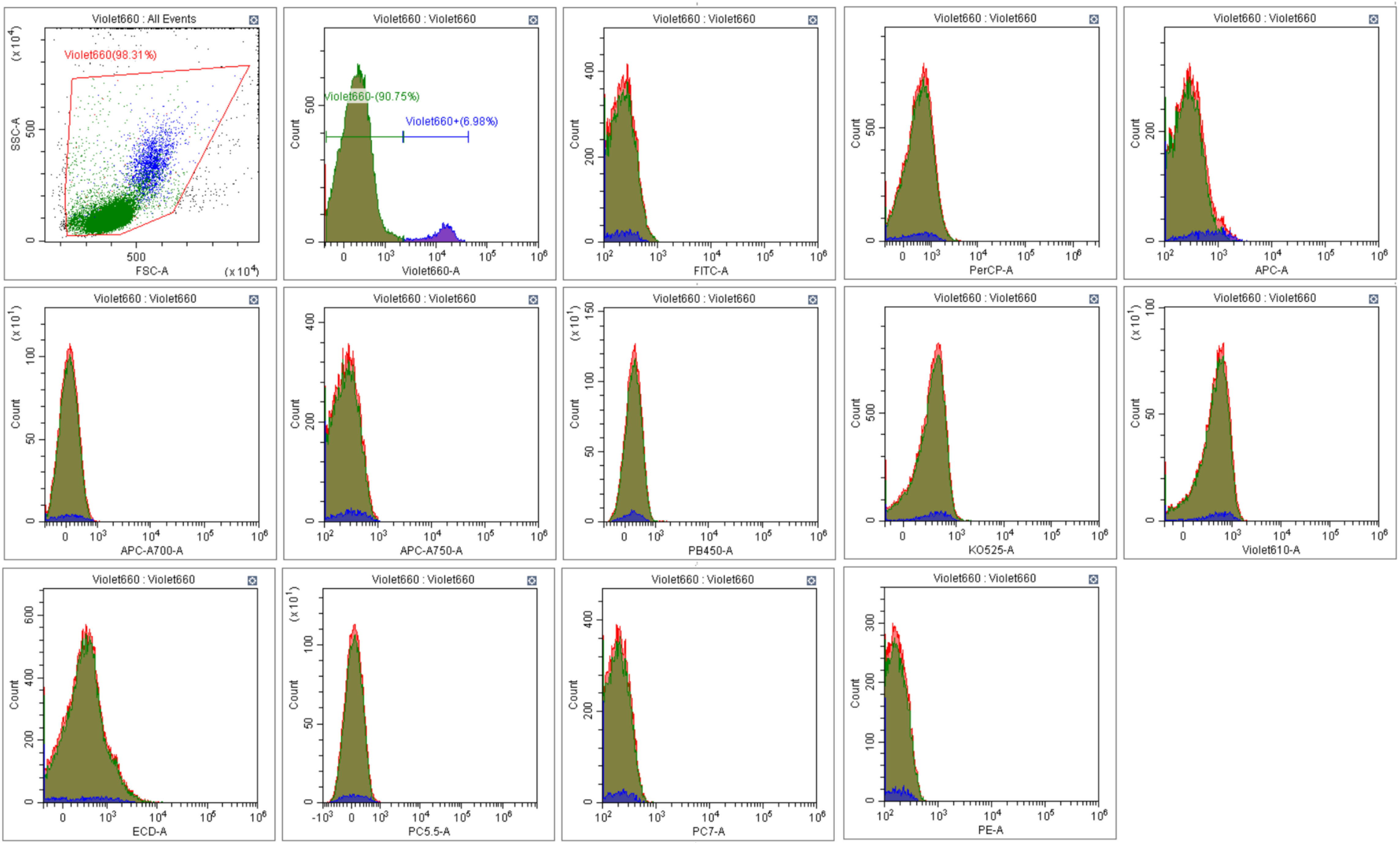

Siglec-1 PE-Texas Red

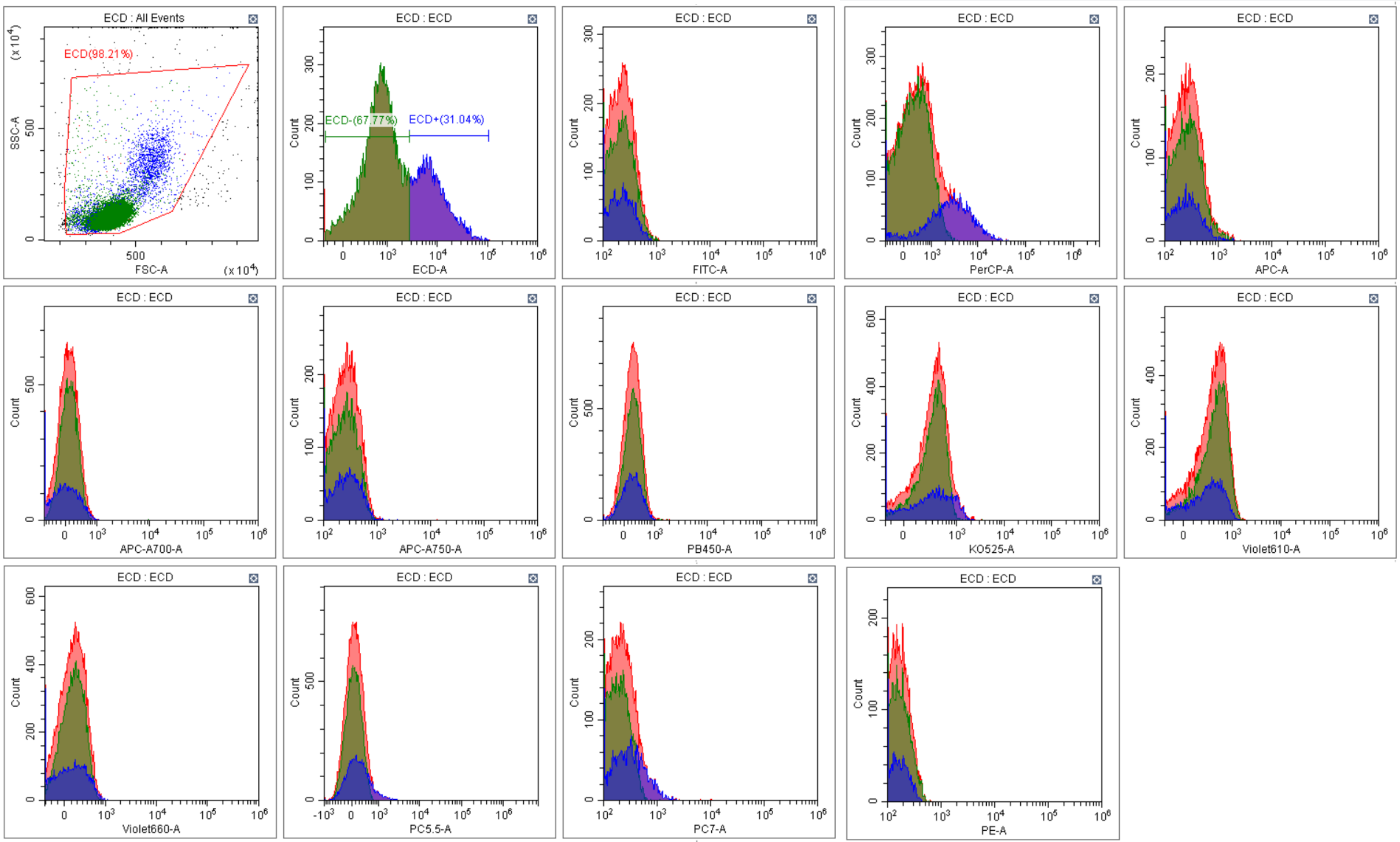

HLA-DR NovaFluorYellow 690

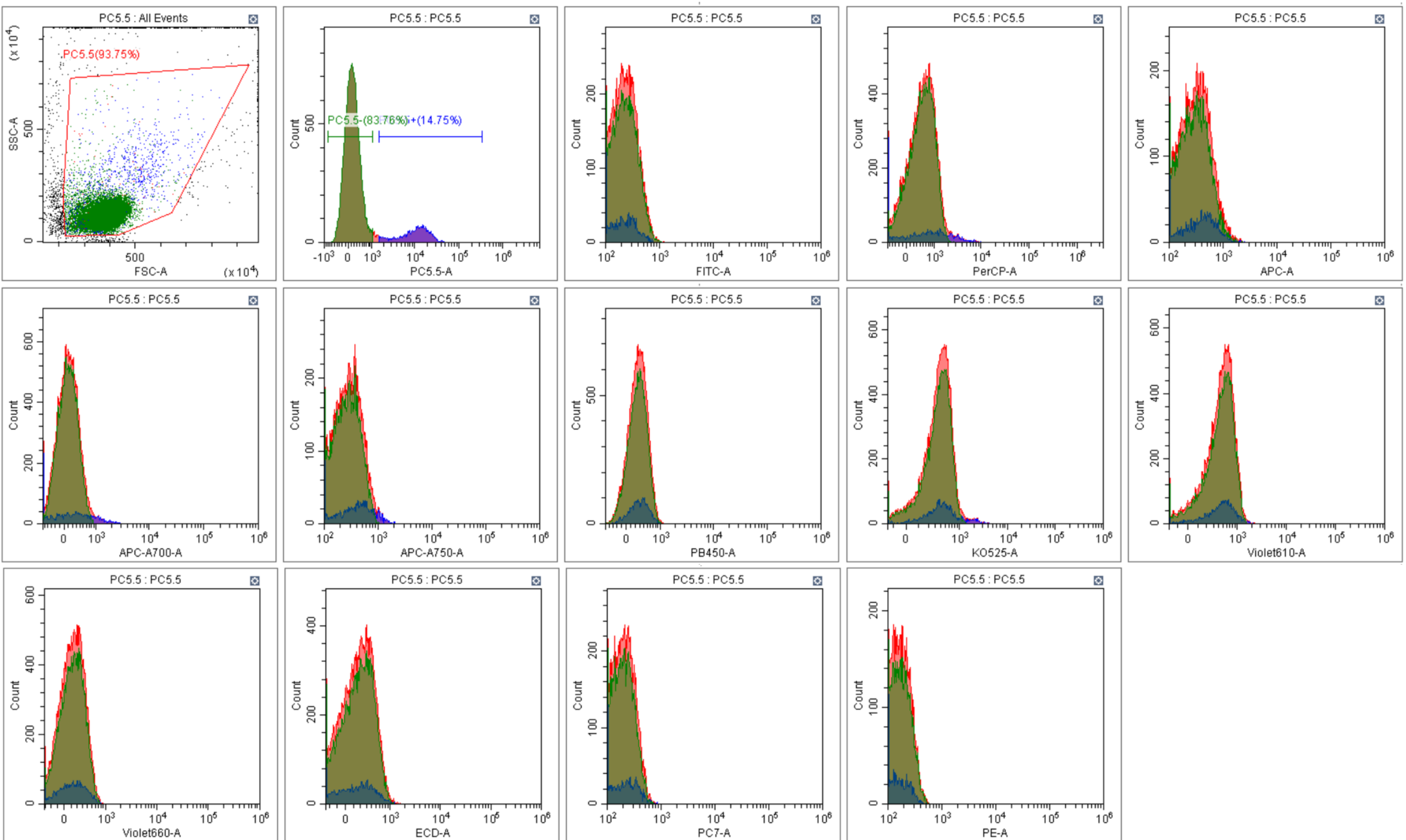

Gal-1 PE-Cy7

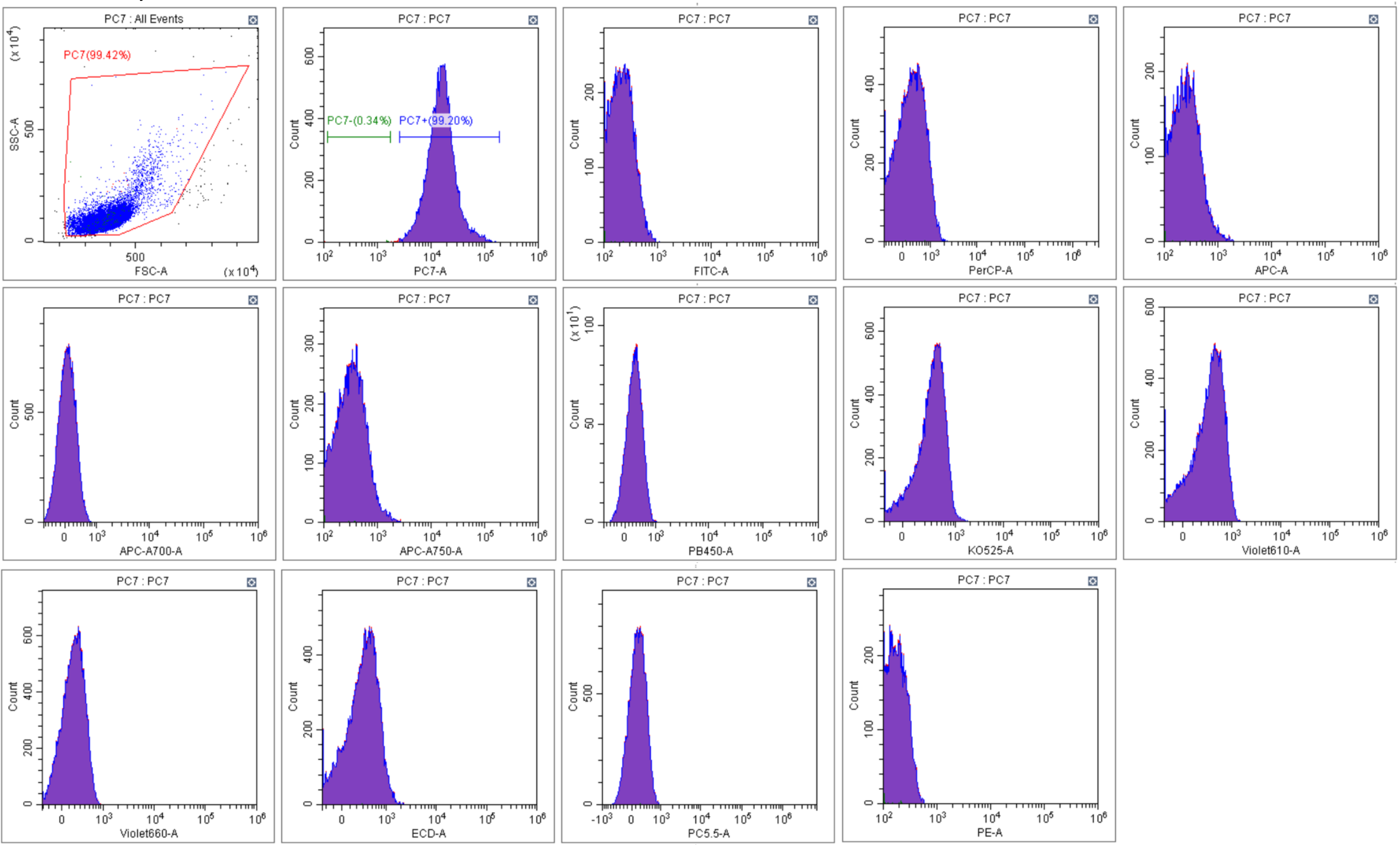
PC7 : All Events
PC7(99.42%)
SSC-A
FSC-A
PC7 : PC7
PC7-(0.34%)
PC7+(99.20%)
Count
PC7-A
FITC-A
PerCP-A
APC-A
APC-A700-A
APC-A750-A
PB450-A
KO525-A
Violet610-A
Violet660-A
ECD-A
PC5.5-A
PE-A
