# Supplementary material for: Identification of immune subsets with distinct lectin binding signatures using multi-parameter flow cytometry: correlations with disease activity in systemic lupus erythematosus
Source: Front Immunol. 2024 May 7;15:1380481. doi: 10.3389/fimmu.2024.1380481 (PMC11106380; doi:10.3389/fimmu.2024.1380481)
Supplement: Supplementary file 2 [file DataSheet_2.docx]

Supplement


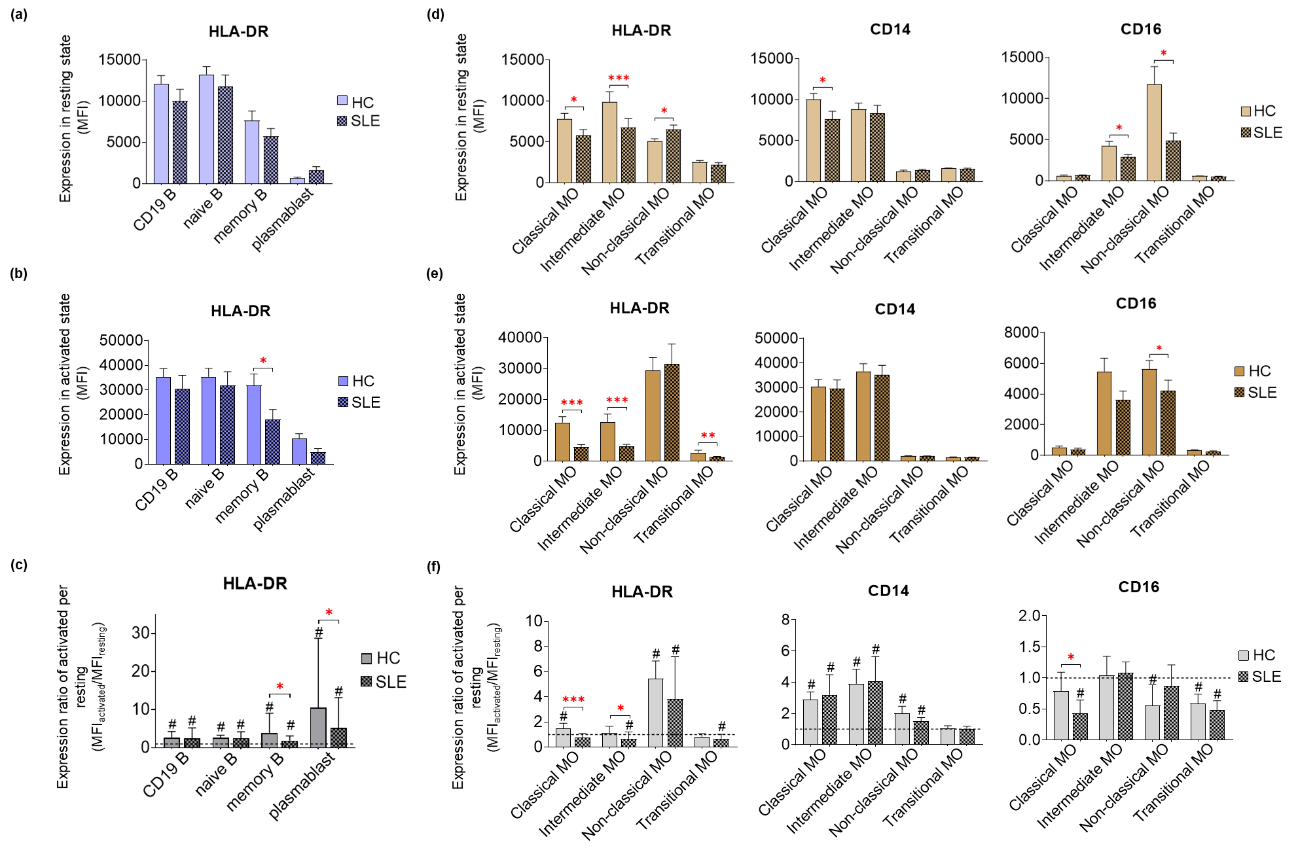


**Supplementary Figure 1.** HLA-DR, CD14 and CD16 expression of manually gated B-cell and monocyte subsets of 18 SLE patients and 18 age- and sex-matched HCs. The binding of fluorochrome-conjugated antibodies was quantified as median fluorescence intensity (MFI) in the (**a** and **d**) unstimulated resting state and (**b** and **e**) after 72-hour activation. In the latter case, B-cells and monocytes in the whole PBMC sample were stimulated with LPS and TLR9 agonist. (**c** and **f**) The lectin binding ratio was calculated between resting and activated cells within each B-cell and monocyte subset (MFI_activated_/MFI_resting_). The symbol “#” marks significant difference (P≤0.05) between resting and activated samples. Data are presented as mean + SEM. *P≤0.05, **P≤0.01, ***P≤0.001.
