# Supplementary material for: Identification of immune subsets with distinct lectin binding signatures using multi-parameter flow cytometry: correlations with disease activity in systemic lupus erythematosus
Source: Front Immunol. 2024 May 7;15:1380481. doi: 10.3389/fimmu.2024.1380481 (PMC11106380; doi:10.3389/fimmu.2024.1380481)
Supplement: Supplementary file 3 [file DataSheet_3.docx]

**
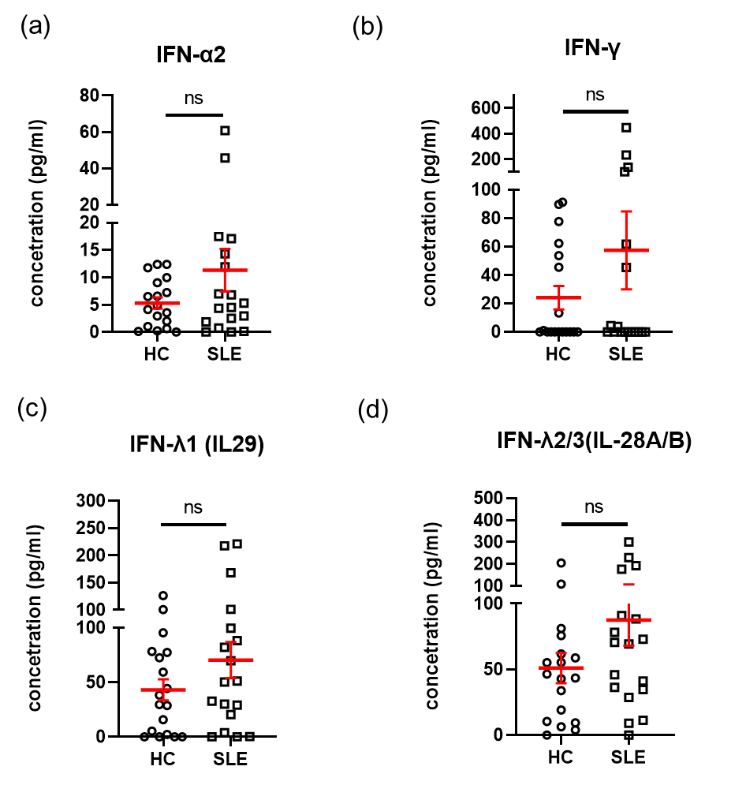
**

**Supplementary Figure 2**

Cytokines measured from the plasma samples of HC (n=18) and SLE (HC=18) patients using Legendplex system. The concentrations are given as pg/mL (mean±SEM) for (**a**) IFN-α2 (**b**) IFN-γ (**c**) IFN-λ1 and (**d**) IFN-λ2 and compared by Wilcoxon test. HC: healthy control; ns: not significant
