# Supplementary material for: Identification of immune subsets with distinct lectin binding signatures using multi-parameter flow cytometry: correlations with disease activity in systemic lupus erythematosus
Source: Front Immunol. 2024 May 7;15:1380481. doi: 10.3389/fimmu.2024.1380481 (PMC11106380; doi:10.3389/fimmu.2024.1380481)
Supplement: Supplementary file 4 [file DataSheet_4.docx]

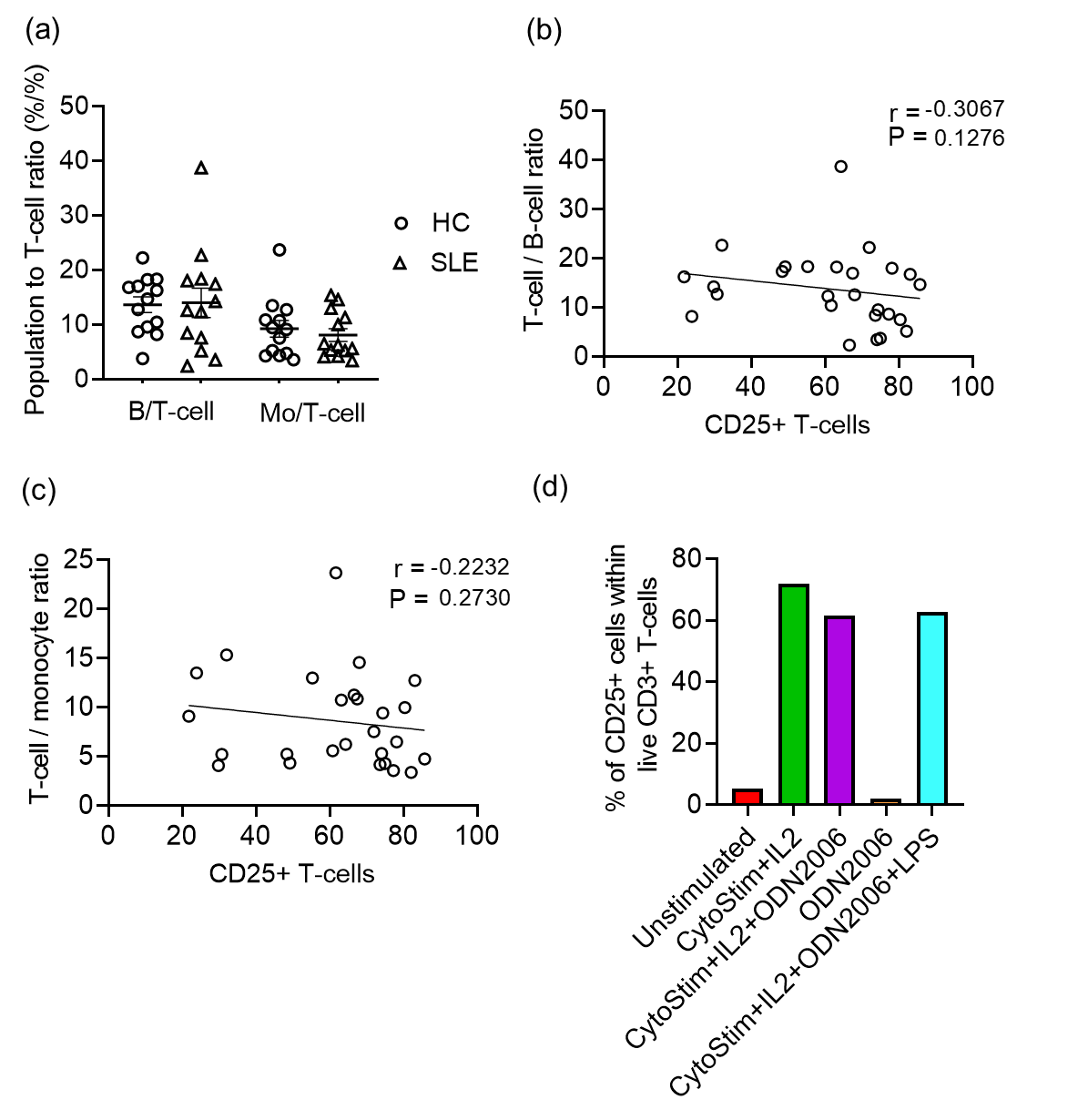


**Supplementary Figure 3.** (**a**) The ratio of B-cell % within living singlets to T-cell % within living singlets, and the ratio of monocyte (Mo) % within living singlets to T-cell % within living singlets were calculated in resting state separately in HCs and in SLE patients. (**b**) Spearman correlation between T-cell/B-cell ratios and the % of CD25+ T-cells within total activated T-cells in the full cohort of HCs and SLEs. (**c**) Spearman correlation between T-cell/monocyte ratios and the % of CD25+ T-cells within total activated T-cells with Spearman correlation coefficients (r) and associated P values. (**d**). PBMC from healthy control was left unstimulated or was stimulated with combinations of CytoStim (1:100), 10 ng/mL human recombinant IL-2, 0.8 μM ODN2006 and 100 ng/mL LPS for 72 hours. Then the % of CD25+ T-cells within live CD3+ T-cells were determined by immune labelling and subsequent flow cytometry.
